# Reduction but no shift in brain activation after arithmetic learning in children: A simultaneous fNIRS-EEG study

**DOI:** 10.1038/s41598-018-20007-x

**Published:** 2018-01-26

**Authors:** Mojtaba Soltanlou, Christina Artemenko, Ann-Christine Ehlis, Stefan Huber, Andreas J. Fallgatter, Thomas Dresler, Hans-Christoph Nuerk

**Affiliations:** 1 Graduate Training Centre of Neuroscience/IMPRS for Cognitive and Systems Neuroscience, 72074 Tuebingen, Germany; 2 Department of Psychology, University of Tuebingen, 72076 Tuebingen, Germany; 3 Leibniz-Institut für Wissensmedien, 72076 Tuebingen, Germany; 4 LEAD Graduate School & Research Network, University of Tuebingen, 72074 Tuebingen, Germany; 5 Department of Psychiatry and Psychotherapy, University Hospital Tuebingen, 72076 Tuebingen, Germany; 6 Center for Integrative Neuroscience, Excellence Cluster, University of Tuebingen, 72076 Tuebingen, Germany

## Abstract

Neurocognitive studies of arithmetic learning in adults have revealed decreasing brain activation in the fronto-parietal network, along with increasing activation of specific cortical and subcortical areas during learning. Both changes are associated with a shift from procedural to retrieval strategies for problem-solving. Here we address the critical, open question of whether similar neurocognitive changes are also evident in children. In this study, 20 typically developing children were trained to solve simple and complex multiplication problems. The one-session and two-week training effects were monitored using simultaneous functional near-infrared spectroscopy (fNIRS) and electroencephalography (EEG). FNIRS measurement after one session of training on complex multiplication problems revealed decreased activation at the left angular gyrus (AG), right superior parietal lobule, and right intraparietal sulcus. Two weeks of training led to decreased activation at the left AG and right middle frontal gyrus. For both simple and complex problems, we observed increased alpha power in EEG measurements as children worked on trained versus untrained problems. In line with previous multiplication training studies in adults, reduced activation within the fronto-parietal network was observed after training. Contrary to adults, we found that strategy shifts via arithmetic learning were not contingent on the activation of the left AG in children.

## Introduction

## Arithmetic learning in adults

Arithmetic learning improves mathematical competence, which is necessary for managing daily transactions and securing job opportunities, among other advantages^1^. The vast majority of our knowledge about the neural underpinnings of arithmetic learning comes from adult studies. Generally speaking, arithmetic learning is characterized by a shift from more effortful and algorithm-based processes towards strategies based mainly on memory and retrieval^2^. Multiplication training studies in adults have illustrated that this strategy shift is accompanied by the reduced activation of the frontal gyri, intraparietal sulcus (IPS), and superior parietal lobule (SPL), and by the increased activation of the left angular gyrus (AG)^3–7^. The change in strategies was also reported in an electroencephalography (EEG) study of complex multiplication training in adults^8^, which revealed an increase in power in theta and alpha frequency bands over parieto-occipital measurement sites.

The fronto-parietal network underlying arithmetic processing includes inferior, middle and superior frontal gyri, which are associated with cognitive processes such as working memory and planning, both useful in mental calculation. According to the triple-code model, the network contains three parietal regions: the IPS, which supports domain-specific magnitude processing of numerals, the SPL, which is associated with domain-general attention processes, and the left AG, which is involved in domain-general verbal processing and the retrieval of information from long-term memory^9^. The retrieval component of the network, however, has been extended to the hippocampus, retrosplenial cortex, and ventromedial prefrontal cortex^10,11^. Moreover, it has been shown that in adults, AG activation varies depending on the learning method used^4^ as well as individual differences^12^, and that the AG may not be engaged by high-level mathematicians during basic calculations^13^. Note that although all of these three areas belong to the parietal lobe anatomically, they are functionally distinguishable during arithmetic processing and learning^9^.

## Activation shifts in children

We focus here on whether the neural activation changes consistently observed in arithmetic learning experiments in adults can be generalized to children, i.e., to the period of life when most people learn arithmetic facts. Neurocognitive learning studies in children are scarce^14,15^, but some information can be drawn from studies on math tutoring, as well as from cross-sectional and longitudinal studies reporting age-related changes.

As in adults, several behavioral studies in children have revealed a strategy shift as an indicator of arithmetic development^16,17^. However, the brain activation changes underlying this shift only partially resemble those found in adults^18–20^. Arithmetic learning consists of two shifts in adults, from frontal areas to parietal areas, and within the parietal lobe, from the IPS and SPL to the left AG^2^. The former shift has been observed in children^21^, but not the latter. Instead, the hippocampus has been proposed as an important transitional region in arithmetic learning in children^22^. A longitudinal study revealed decreased activation in the bilateral dorsolateral prefrontal cortex, the left SPL, and the right parieto-occipital cortex, as well as increased activation of the bilateral hippocampus over the course of one year of school education in 7- to 9-year-old children^22^. Qin and colleagues^22^ reported a correlation between the increasing use of fact retrieval strategies and hippocampal connectivity to the bilateral dorsolateral prefrontal cortex and left IPS, but not to the left AG. The authors suggested that the medial temporal lobe, including the hippocampus, plays a critical transient role in arithmetic learning in children, but not in adults^22^. In line with this finding, a one-on-one math tutoring study in third-grade children reported changes in the morphometry of the hippocampus and its connectivity with frontal regions as the best predictor of improvement in arithmetic problem-solving^23^.

A cross-sectional study of simple multiplication performance in children from grades 2 to 7 showed grade-related decreases in inferior frontal gyrus (IFG) activation – similar to arithmetic learning in adults – and increases in left middle temporal gyrus (MTG) activation, accompanied by increased dependence on retrieval strategies as a function of age^21,24^. Therefore, although systematic standardized training studies have not yet been carried out, the available studies suggest that learning-related changes in activation in children may be different than in adults^25^. In particular, however, the current literature on children’s learning does not show increased (or less deactivated) AG activation, which characterizes retrieval learning in adults. Note that recent empirical findings^26^ and theoretical models^11^ have suggested the engagement of the hippocampus in retrieval processes in adults as well. Differences between adults’ and children’s learning have been also observed in EEG studies as well^18–20^. For instance, enhanced theta power during arithmetic processing was reported in adults as opposed to children, which was interpreted as reflecting the higher working memory capacity of adults^19^. We conclude that while the reduction in activation of some areas of the fronto-parietal network seems similar between adults and children, increases in activation in regions related to retrieval strategies differ between adults and children.

These findings, however, cannot be directly transferred to children’s arithmetic learning in general. In a tutoring study, children received training within several different mathematical domains and learned various problem-solving strategies, and they were tested only on single-digit addition problems^23^. Moreover, children underwent a tutorial training based on their weaknesses; therefore, specific factors (e.g., numerical training) and nonspecific factors (e.g., increased motivation) due to the one-on-one training cannot be disambiguated. Longitudinal (and even more so, cross-sectional) studies on arithmetic learning are limited by another problem. Arithmetic learning throughout childhood is strongly associated with brain maturation. Therefore, it is difficult to determine whether activation changes are truly associated with the arithmetic learning process in school, or are rather a byproduct of maturation of the whole brain. Altogether, and in light of a recent review paper on the neural correlates of arithmetic development in children that demonstrates the incompleteness of our knowledge on the subject^14^, it remains essential to investigate brain activation changes due to carefully controlled arithmetic training in children within a relatively narrow age range.

## The present study and its objectives

To examine learning processes in children, we used multiplication, the operation most frequently investigated in arithmetic learning studies in adults. To the best of our knowledge, brain activation changes after multiplication training have not been investigated in children so far. The present study aimed to explore the brain activation changes related to simple and complex multiplication learning in typically developing children. In order to evaluate the training-induced changes, we used simultaneous functional near-infrared spectroscopy (fNIRS) and EEG as pre- and post-training measurements in an ecologically valid setting that allowed children to perform small movements and provide answers manually, as in school and other learning situations^27^. While fNIRS is an established method for investigating brain activation changes in children in some fields, such as language processing, it has rarely been used in the field of numerical cognition. Conversely, oscillatory EEG is an established method in the field of numerical cognition, but has been used mainly in adults. Therefore, applying these two methods simultaneously aids in interpreting previous results from each of the two perspectives. Furthermore, the combination of these two neuroimaging methods increases the construct validity of the findings and allows for a multi-level assessment of underlying neurobiological processes, measuring both the regions involved and neural network dynamics, to produce both direct and indirect signals of brain activity. Moreover, in addition to behavioral findings, EEG might be helpful to indicate strategy use^28^.

Regarding EEG, it has been shown that cognitive processes lead to event-related synchronization (ERS)/desynchronization (ERD). ERS and ERD concern all power in the signal, including both phase-locked and non phased-locked components, providing quantifiable measures of brain dynamics^29^. While ERS represents increased power, ERD represents decreased power during mental processing compared to rest. Previous studies have found that cognitive processes result in brain oscillation changes in theta and alpha bands^30^. Therefore, similar to most of the previous studies in the field of numerical and arithmetic processing^31–34^, these two frequency bands were investigated in the present study. Because we were interested in learning-related brain activation changes in a natural setting, we employed a self-paced paradigm in a block-design experiment. Therefore, ongoing EEG was recorded during blocks of mental calculation and further analyzed by the ERS/ERD method.

Based on previous findings from studies in children and adults, we hypothesized a strategy shift from slow, procedural strategies to fast, compact procedural and retrieval strategies after training, which would lead to more efficient responses, i.e., shorter response times and fewer errors^14,35^. This strategy shift is also linked to brain activation changes in the fronto-parietal network. We expected reduced activation within the frontal gyri, IPS, and SPL. Regarding activation of the left AG, while multiplication training studies in adults have reported increased activation^2^, some studies have observed a decrease in AG activation concurrent with an increase in expertise^13,36^, and developmental studies have reported no change in activation^25^. Therefore, no direct hypothesis was obtainable regarding the left AG. Note that because fNIRS is limited to measuring only cortical activation, we could not make any hypotheses about activation changes in deep brain structures such as the hippocampus. Regarding EEG oscillation, we initially expected an increase in theta and alpha power after training, as shown in studies of multiplication learning in adults^8^, marking a reduction in cognitive demand^30^. An arithmetic processing study in children^27^, however, reported greater theta ERS during complex multiplication than during simple multiplication. Reasoning that training makes problems arbitrarily simpler, we expected a reduction in theta power after training in the present study, in contrast with adults. A few oscillatory EEG studies in children, which have shown neurophysiological differences between children and adults, support this hypothesis^18–20^.

Because studies in adults have revealed similar shifts in brain activation patterns during the course of training^6^ and after several sessions of training^3^, we aimed to measure the effects of a single session and of seven sessions, or two weeks, of multiplication training in children. In accordance with above-mentioned studies, similar brain activation changes were expected after each training period. Furthermore, to examine transfer effects from multiplication training to basic arithmetic ability^7^, a modified math ability test was used^37^. As in prior literature^8,31^, we also measured strategy changes by directly asking children how they solved the problems, before and after the training sessions.

## Materials and Methods

### Participants

26 typically developing children from grade 5 participated in the study. After excluding participants for technical reasons, noisy data, or quitting training (one participant), a total of 20 children (8 girls; 11.1 ± 0.5 years old) were included in the analyses (see details in SI). All children were right-handed and had normal or corrected-to-normal vision with no history of neurological or mental disorders. Children and their parents gave written informed consent and received an expense allowance for their participation. All procedures of the study were in line with the latest revision of the Declaration of Helsinki and were approved by the ethics committee of the University Hospital Tuebingen.

### Materials

16 simple and 16 complex multiplication problems were used in the present study (cf. SI, Table S1). Half of the problems in each set were allotted for training, and the other, closely matched half were used as untrained problems, resulting in four conditions: trained simple, untrained simple, trained complex, and untrained complex. The sets were matched based on the sizes of the operands and results, and the parity of the operands and results, separately for simple conditions and complex conditions. The matching was supported by the finding of non-significant differences between trained and untrained conditions in pre-training session, *ts* < 1.32, *ps* > 0.20. 16 simple problems (e.g., 4 × 6) included two single-digit operands (range 2–9) with two-digit solutions (range 12–40). 16 complex problems (e.g., 7 × 13) included a double-digit (range 12–19) multiplied by a single-digit operand (range 3–8), with double-digit solutions (range 52–98). The sequence of small and large operands within the problems was counterbalanced. Problems with ones (e.g., 9 × 1), commutative pairs (e.g., 3 × 4 and 4 × 3) or ties (6 × 6) were not used.

### FNIRS

FNIRS data were collected with the ETG 4000 Optical Topography System (Hitachi Medical Corporation, Tokyo, Japan) using two wavelengths of 695 ± 20 nm and 830 ± 20 nm to measure the absorption changes of oxygenated (O_2_Hb) and deoxygenated (HHb) hemoglobin, according to the modified Beer-Lambert law. The data were recorded with a 10 Hz sampling rate, and the fixed inter-optode distance was 30 mm. Using a 3 × 5 arrangement of the optodes (8 emitters, 7 detectors) in an elastic combined fNIRS-EEG cap (Brain Products GmbH, Herrsching, Germany), 22 measurement channels were shaped over each hemisphere. The correspondence of the fNIRS channels to the underlying cortical areas was estimated based on a virtual registration method^38–40^ and labeled according to the automatic anatomical labeling (AAL) atlas^41^ in SPM software (http://www.fil.ion.ucl.ac.uk/spm). In this method, virtual holders are constructed on spherical phantoms and registered on synthetic heads and brains. Thereafter, the registered positions are normalized to the Montreal Neurological Institute (MNI) template. Repeating these procedures leads to a highly plausible estimation of spatial coordinates^40^. This method shows only a small spatial registration error within an acceptable limit when tested for the human brain^39^. Therefore, it is suitable for group analysis of fNIRS data, even when participants’ structural MRI scans are not available.

### EEG

EEG data were recorded with a 32-channel DC-amplifier and the software Vision Recorder (Brain Products, Munich, Germany). 21 scalp EEG electrodes, attached to the combined fNIRS-EEG cap, were used for EEG data collection. Given the fixed optode distances, EEG electrodes were placed according to the extended international 10–20 system^42,43^. In addition, eye movements were recorded using electrooculography (EOG) in one electrode placed below the right eye. The ground electrode was placed frontally on AFz and the online reference electrode fronto-centrally on FCz. Electrode impedance was kept below 20 kΩ. Data were digitized at a rate of 1000 Hz with an online bandpass filter of 0.1–100 Hz.

### Neuropsychological tests

In order to assess the homogeneity of the sample, IQ and memory abilities were measured (see SI, Table S3). Two subtests (similarities and matrix reasoning) of the German Wechsler IQ test^44^ were utilized to assess intelligence. Furthermore, four components of memory, namely verbal short-term and working memory, and visuospatial short-term and working memory, were assessed^45^. The letter span test was used to measure verbal memory capacity, while the Corsi block tapping test^46^ was used to assess visuospatial memory capacity^47^. In our verbal short-term memory task, children were asked to recall spoken sequences of letters (one letter per second). The test started with sequences of two letters, which were increased by one letter if the child correctly recalled at least two out of three sequences. In the short-term visuospatial memory task, the child was asked to point to cubes in the same order as the experimenter. The procedure was the same as in the letter span test. These forward spans were considered to represent short-term memory, while backward spans were considered to show working memory. Moreover, a modified math ability test^37^ was used before and after training to assess the effects of multiplication training on other basic arithmetic problem-solving.

Furthermore, a brief, self-developed strategy questionnaire was used before and after training. Because of time limitations, we could not ask children about their strategy use after each multiplication problem, but this questionnaire was developed to elicit some information about possible strategy shifts. The questionnaire consisted of eight multiplication problems, two from each set, for a total of four different matched lists. There was no time limit for responding to the problems. After responding to each trial, children reported how they arrived at the solution. According to the child’s report, experimenters categorized each strategy as retrieval, procedural, or “other”^31^. The inter-rater reliability as indicated by Cohen’s kappa was 0.80.

### Measurement procedure

**Figure 1:**
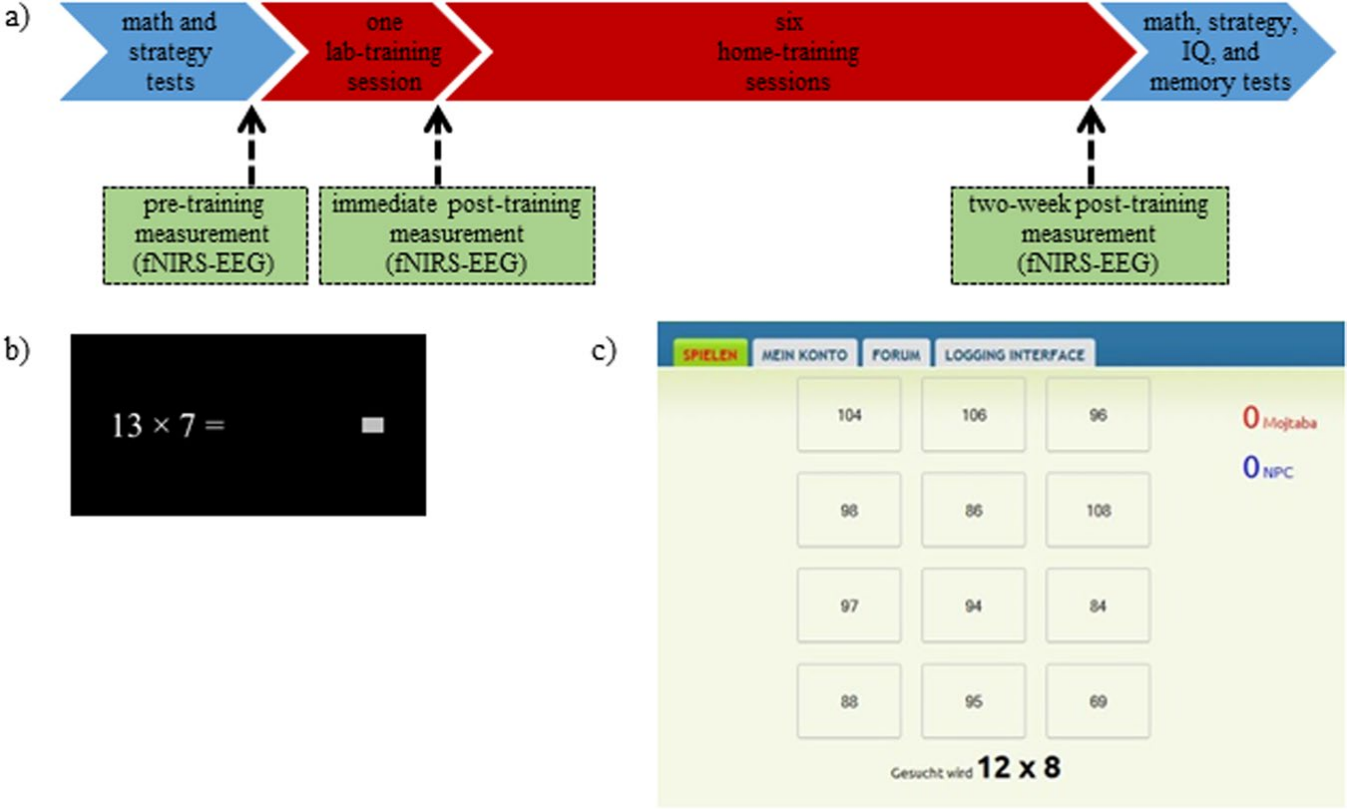
(**a**) The Experiment: pre-training measurement was conducted in the first day before any training. A first post-training measurement was conducted after one session of training using an online learning platform in the first day. A second post-training measurement was conducted after two weeks of training. (**b**) After responding, pressing the gray box presented the next problem. (**c**) Online learning platform: in competition with a computer, children had to select the correct answer out of 12 possible choices.

In a within-subject experiment, performance and brain activation of children were measured during multiplication problem-solving at three measurement times (cf. Fig. 1a): before training, after one session of training (one-session effect), and after seven sessions of training (two-week effect). First, children performed the math ability test and strategy questionnaire. The experiment was conducted after four practice trials in a light-attenuated room. Problems were presented on a touch screen and children had to write their answers as quickly and accurately as possible and then click on a gray box, presented on the right side of the screen, to continue (see Fig. 1b). The written response was not visible, to avoid any further correction and to encourage children to calculate mentally. The problems of each condition were presented in four blocks of 45 s, each followed by 20 s of rest. The sequences of the blocks and of problems within the blocks were pseudo-randomized. Whenever the total number of trials within a condition was reached, the same problems were presented again after randomization. No feedback was given during the experiment. The design was self-paced with a limited response interval of 10 s for simple and 30 s for complex problems. Therefore, due to inter-individual differences, the number of solved problems varied between children. The inter-trial interval was set to 0.5 s. After the pre-training session, children performed one session of approximately 25 minutes of interactive training (see below). In order to investigate one-session training effects, the first post-training measurement was performed directly afterward. The whole procedure lasted approximately 2.5 hours. Thereafter, six similar training sessions were performed at home over the course of two weeks. Children were measured again in order to evaluate two-week training effects (cf. Fig. 1a). In this second post-training session, the math ability test and strategy questionnaire were administered again, along with the other neuropsychological tests. The problems, but not the sequence of the blocks or problems, were identical for each condition in pre-training and post-training sessions. The experiment was run using Presentation® software version 16.3 (Neurobehavioral Systems Inc., www.neurobs.com).

### Interactive training procedure

Training was done using an online learning platform^48–50^, which allowed for at-home training. One training session (including only trained simple and trained complex conditions) was performed in the lab and six at-home sessions were performed by children during a two-week interval. The problems of each condition were randomly repeated six times in each training session. Note that several children did not finish some sessions during the training at home, and after some time came back and did the training. Since they could not continue the task from the trial on which they had quit before, they instead needed to start an entire session again. Therefore, some training sessions included more than six repetitions of the problems (cf. SI, Table S2). Each problem was individually presented along with 12 different choices including the correct solution (see Fig. 1c). A similar method has been already used in adults and children^20^. Response intervals of simple problems ranged randomly between 4 and 10 s, jittered by 0.6 s, and of complex problems between 10 and 30 s, jittered by 2 s. Whenever the child did not respond within the response interval, the computer screen displayed the correct solution. Training was interactive in the sense that children had to compete with the computer. To provide feedback about the performance and to increase motivation, the scores of the child and computer were shown on the right side of the screen. Both child and computer received one point for each correct answer and one point was deducted for each incorrect answer. The problem was presented until the child or computer responded correctly. In order to create a more realistic competition, the computer responded incorrectly in 30% of the problems. Children were instructed to solve the problems as quickly and accurately as possible.

### Analysis

#### Behavioral

Written responses by children were read out with the help of the non-commercial RON (ReadOutNumbers) program (Ploner, 2014). Response times (RTs) were defined as the time interval from problem presentation to children’s clicking the gray box. Only median RTs for correct responses (78.7% of problems across all measurement times) were included in the analyses. The error rate was defined as the proportion of incorrect or missing responses to the total number of presented trials. The inverse efficiency score represents the median RT divided by the percentage of correctly solved problems^51^. Smaller inverse efficiency scores indicate more efficient performance. Separated repeated measures analyses of variance (rmANOVAs) were conducted to investigate one-session and two-week training effects on median RTs, arcsine-square-root-transformed error rates^52^, and inverse efficiency scores. The 2 × 2 × 2 rmANOVA comprised within-factors of measurement time (pre- versus post-training), training (trained versus untrained), and complexity (simple versus complex). Further rmANOVAs and paired *t*-tests were conducted separately for simple and complex multiplication. Note that because inverse efficiency combines RTs and accuracy of responses, and due to space limitations, only inverse efficiency scores are explained in the following sections. Separate results for RTs and errors on the training effect are reported in SI (cf. SI, Figs S2 and S3).

In order to uncover transfer effects from multiplication training to other arithmetic operations, a 2 × 4 rmANOVA consisting of measurement time (pre- versus post-training) and operation (addition, subtraction, multiplication, and division) as within-factors was conducted. In addition, paired *t*-tests were conducted separately on each operation. To determine the effect of multiplication training on strategy use, paired *t*-tests were conducted on retrieval and procedural strategies separately. The analysis was completed using SPSS version 23.0 (IBM SPSS Statistics for Windows). The effect sizes of *t*-tests were also calculated^53^.

#### FNIRS

Continuous changes in the concentration of O_2_Hb and HHb were recorded for all channels during the measurements. These changes occur through neurovascular coupling in response to cortical activation. Data were analyzed with custom MATLAB routines (The MathWorks, Inc., USA). The continuous signals were bandpass filtered with 0.008–0.09 Hz in order to remove long-term drift of baseline, Mayer waves, and high-frequency cardiac and respiratory activities^54–56^. It has been shown that fNIRS signals, like other blood-related brain measures, are low-frequency oscillations, detectable mainly between 0.01 and 0.1 Hz^56,57^. Applying a more liberal low-pass filter of 0.7 Hz^58^ led to nearly the same results, although decreased activation at the left AG did not survive correction for multiple comparisons (see the Results section). Remaining noisy channels were interpolated using the average of surrounding channels for each participant. To address possible motion artifacts, particularly likely in children, and to reduce non-evoked systemic influences^54,59^, we used the correlation-based signal improvement (CBSI) method^60^. This CBSI time course, which is calculated based on the negative correlation between concentrations of O_2_Hb and HHb, was used for further analysis. The optimal correction approach, however, is data-dependent^61^. The general linear model (GLM) analyses were performed for each participant and each condition. The model-based signal, which was a boxcar regressor indicating the beginning and 40 s of each block, convolved with the hemodynamic response function (HRF), was used for further analysis^54^. The last 5 s of the blocks were excluded from analysis as signal quality got noisier towards the end of the blocks. Thereafter, means of least-square linear regression were applied to calculate the beta-values of each channel.

#### ROI analysis

Similar to our behavioral data analysis, a 2 × 2 × 2 rmANOVA comprising the within-factors of measurement time (pre- versus post-training), training (trained versus untrained), and complexity (simple versus complex) was conducted to find the one-session and two-week training effects separately in each region of interest (ROI). To this end, and based on the topography of brain activation changes in our recent fNIRS study in children^27^, we defined four ROIs within the fronto-parietal network, including four channels for each: left and right frontal, and left and right parietal regions (see SI, Fig. S1 and Table S4). The frontal network comprised middle frontal gyrus (MFG) and IFG, and the parietal network comprised IPS, SPL, and AG (see SI, Table S4). Furthermore, in the case of a significant interaction, additional rmANOVAs, and paired *t*-tests were conducted. The significance level was 0.05.

#### Channel analysis

Since there are distinct networks within the parietal lobe^9^, similar 2 × 2 × 2 rmANOVAs were conducted over the channels within each parietal ROI. In the case of a significant interaction, additional rmANOVAs and paired *t*-tests were conducted.

#### Whole measurement area analysis

In order to examine the effects of training on the whole measurement area, multiple paired *t*-tests between trained versus untrained conditions were calculated for each channel. To this end, the contrasts of trained versus untrained conditions after one session of training were compared with the contrasts of trained versus untrained conditions in the pre-training measurement (for instance in complex multiplication: [trained complex _post-training_ − untrained complex _post-training_] − [trained complex _pre-training_ − untrained complex _pre-training_]). These contrasts indeed demonstrate the interaction of measurement time (pre- versus post-training), and training (trained versus untrained) separately for simple and complex conditions. The same contrasts were calculated between pre-training and post-training measurements after two weeks to evaluate the two-week training effect. The significance level was 0.05, and correction for multiple comparisons was performed using the Dubey/Armitage-Parmar (D/AP) method^62^. The D/AP method is among the stepwise modified Bonferroni procedures that consist of readjusting the level of significance for the individual test while taking into account autocorrelations in the data. Among several adjustment methods, D/AP is the only procedure which fully considers the correlations of the data^62^. This procedure is well suited to the analysis of fNIRS data, due to the typically strong correlations between neighboring fNIRS channels. Furthermore, Bayesian analysis was conducted by means of JASP (Version 0.8.1.1; JASP Team, 2016).

#### EEG

EEG data were analyzed using the Brainstorm toolbox^63^, a documented and freely available software package (http://neuroimage.usc.edu/brainstorm). EEG signals of 21 electrodes were offline rereferenced to an average reference and filtered using a bandpass of 0.1–40 Hz. Based on the EOG signal, eye movement artifacts were detected and removed from the EEG signals using Signal Space Projections (SSP). SSP does not depend on additional reference sensors to record the disturbance fields. Instead, SSP assumes different spatial distributions for the magnetic field generated by the sources in the brain and those generated by external noise sources^64^. Therefore, SSP detects and removes artifacts that have more complex frequency pattern but are well-defined.

In order to assure sufficient signal quality, we used the following steps. We asked and reminded children to avoid unnecessary movements. One experimenter was recording every unexpected reaction from the child (e.g., coughing, sneezing, verbal movement, and questions), enabling us to check and exclude disrupted sections of the recording whenever necessary. After visual inspection, we excluded any samples that were overall very unstable and noisy. Most of the eye movement artifacts have slow oscillations, which interfere with delta band activation and are represented in prefrontal regions. Since in the present study our focus was on theta and alpha bands, there may be negligible noise in these frequency bands. Still, we applied the SSP method to subtract out any remaining movement noise in the signal and visually inspected the distribution of the eye movements on the scalp. Moreover, because the present study was a within-subject design, we subtracted the ERS/ERD of the untrained conditions from the trained conditions, which led to subtracting out part of the remaining noise, particularly noise related to arm/hand movements due to writing the answers.

In the next step, epochs were extracted with a block duration of 45 s and rest duration of 20 s. The power spectral density (PSD) was calculated for the theta (4–7 Hz) and alpha (8–12 Hz) bands, and individually averaged for each condition and measurement time. To measure the cortical activation and functional changes of brain activity^65^, ERS/ERD was calculated. The percentage values of ERS/ERD were calculated by this expression: ERS/ERD % = (PSD of activation − PSD of rest)/PSD of rest × 100^65^. The ERS/ERD are quantitative measures of brain dynamics^66^. Because of the sensitivity of the EEG signal to several factors such as individual differences, age^67^, and brain volume^68^, the analysis of changes in the EEG signal is more reliable than the absolute power of the frequency band^65^. ERS is indicated as larger power spectral density (PSD) of a condition than at rest, which leads to a positive value, while ERD is indicated as a negative value because the PSD of a condition is smaller than at rest.

#### ROI analysis

Based on the topography of frequency oscillation in previous studies^8,69^, six ROIs within the fronto-parietal network were defined: left, right, and middle fronto-central, left, right, and middle occipito-parietal regions (see SI, Fig. S1, and Table S4). Within theta and alpha frequency bands, a 2 × 2 × 2 rmANOVA was conducted for each ROI separately. In each step, in the case of significant interaction, additional rmANOVAs and paired *t*-tests were conducted separately for simple and complex multiplication. The significance level was 0.05.

#### Whole measurement area analysis

In order to examine the effects of training on the whole measurement area, similar to fNIRS data analysis, paired *t*-tests between the contrasts in pre- and post-training sessions were calculated (see above), showing the interaction of measurement time (pre- versus post-training), and training (trained versus untrained) separately for simple and complex conditions. The significance level was 0.05 uncorrected.

## Results

### Behavioral

#### One-session training

**Figure 2:**
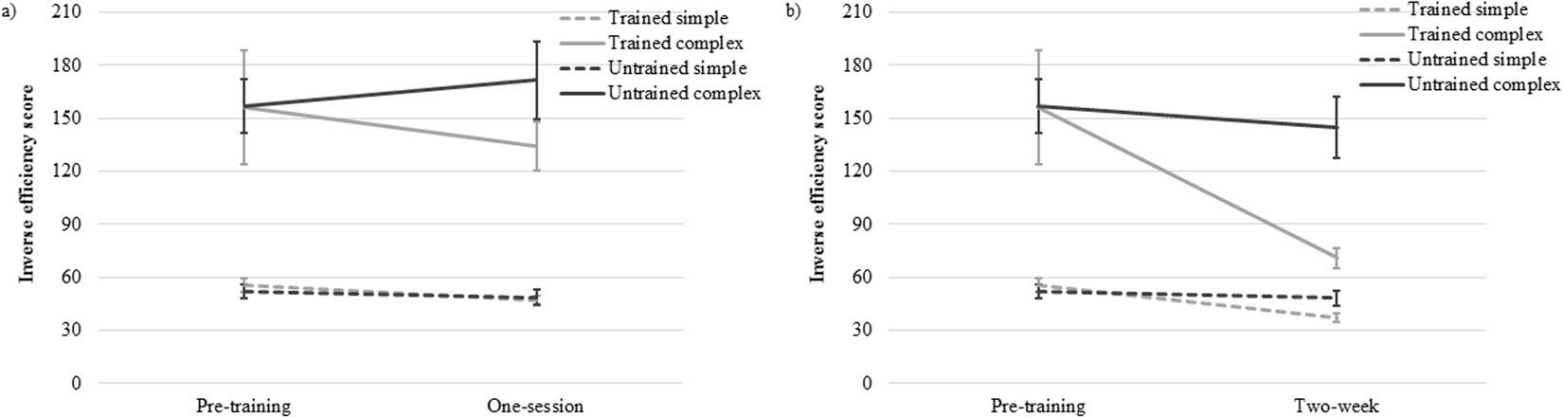
Inverse efficiency score changes. (**a**) one-session training effect, and (**b**) two-week training effect. Smaller inverse efficiency scores indicate more efficient performance. Error bars reflect SEs.

With one session of training, inverse efficiency scores indicated a significant main effect of complexity [*F*(1,19) = 55.17, *p* < 0.001, η^2^ = 0.74], showing that performance was better on simple than complex multiplication problems overall (see Fig. 2a). The other main effects and interactions did not reach statistical significance after just one session [*Fs*(1,19) < 2.2, *ps* > 0.15, η^2^ < 0.11].

#### Two-week training

In the two-week training condition, inverse efficiency scores revealed significant main effects of measurement time, training, and complexity [*Fs*(1,19) > 7.4, *ps* < 0.013, η^2^ > 0.27]. A significant interaction of measurement time × training showed that training led to an additional improvement in trained compared to untrained problems [*F*(1,19) = 6.45, *p* = 0.02, η^2^ = 0.25]. This interaction was explored by two separate 2 × 2 rmANOVAs for simple and complex problems. In the simple multiplication condition, a significant main effect of measurement time revealed that inverse efficiency scores improved after two weeks of training [*F*(1,19) = 18.16, *p* < 0.001, η^2^ = 0.49]. Then, a significant interaction of measurement time × training showed that children improved further on trained than untrained simple problems [*F*(1,19) = 13.79, *p* = 0.001, η^2^ = 0.42] (cf. Fig. 2b). The main effect of training was not significant in the simple multiplication. With respect to complex multiplication, significant main effects of measurement time and training were observed [*Fs*(1,19) > 5.7, *ps* < 0.027, η^2^ > 0.23]. An interaction of measurement time × training showed a significant training effect in trained complex multiplication compared to untrained complex problems [*F*(1,19) = 4.75, *p* = 0.042, η^2^ = 0.20] (cf. Fig. 2b).

A marginally significant interaction of measurement time × complexity [*F*(1,19) = 4.02, *p* = 0.059, η^2^ = 0.18], a significant interaction of training × complexity [*F*(1,19) = 6.64, *p* = 0.018, η^2^ = 0.26], and a marginally significant interaction of measurement time × training × complexity [*F*(1,19) = 3.14, *p* = 0.093, η^2^ = 0.14] were also observed.

### FNIRS

#### Results of ROI analysis

**Figure 3:**
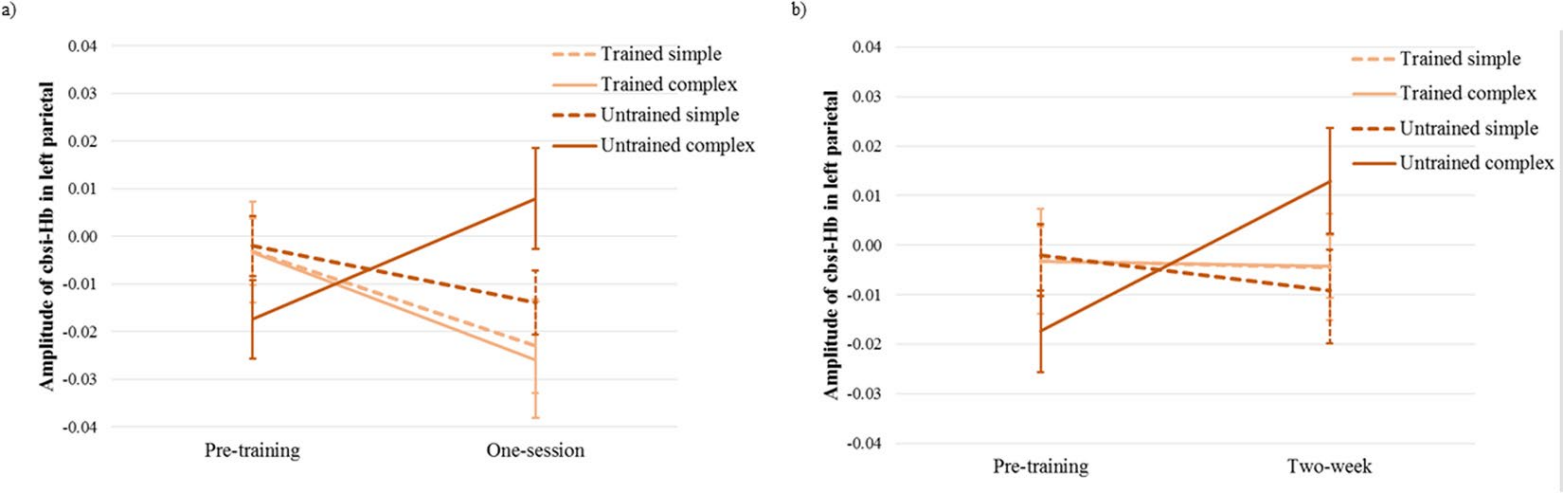
Brain activation changes in the left parietal region as (**a**) one-session training effect, and (**b**) two-week training effect (the lines representing trained simple and trained complex are almost over each other). Error bars reflect SEs.

*One-session training*. In the absence of one-session behavioral improvement, the rmANOVA on the ROIs revealed a significant one-session training effect in the left parietal lobe. We observed a significant interaction of measurement time × training [*F*(1,19) = 6.33, *p* = 0.021, η^2^ = 0.25]. To explore this interaction, two separate 2 × 2 rmANOVAs for simple and complex conditions were conducted. In simple conditions, a significant main effect of measurement time demonstrated decreased activation of the left parietal lobe after the training [*F*(1,19) = 5.97, *p* = 0.024, η^2^ = 0.24] (see Fig. 3a). No other significant effect was found in simple conditions.

In complex conditions, a significant interaction of measurement time × training showed decreased activation during trained complex multiplication problems, while activation increased in the left parietal lobe during untrained complex multiplication problems [*F*(1,19) = 8.25, *p* = 0.01, η^2^ = 0.30] (see Fig. 3a). Further analysis revealed a significant difference between trained and untrained complex conditions only after training [*t*(19) = -2.4, *p* = 0.027, *d* = 0.54], but no significant difference before training and over time. The main effects of measurement time and training were not significant. Moreover, a marginally significant interaction of measurement time × training × complexity was found in the left parietal lobe [*F*(1,19) = 3.74, *p* = 0.068, η^2^ = 0.16]. No significant effect of one-session training was observed in other ROIs.

*Two-week training*. Similar to the one-session training effect, the rmANOVA on ROIs revealed significant effects of two-week training only in the left parietal lobe. A significant interaction of measurement time × training × complexity was observed [*F*(1,19) = 8.40, *p* < 0.01, η^2^ = 0.31]. Further rmANOVA analyses, conducted separately for simple and complex conditions, revealed no significant training effect in simple conditions.

In complex conditions, a significant interaction of measurement time × training showed decreased activation in the trained condition, while increased activation was observed in the left parietal lobe in the untrained condition [*F*(1,19) = 5.53, *p* = 0.03, η^2^ = 0.23] (see Fig. 3b). Further analysis demonstrated only significantly increased activation in the untrained complex condition [*t*(19) = 2.57, *p* = 0.019, *d* = 0.58], but no significant decrease in the trained complex condition over time. The main effects of measurement time and training were not significant. No significant two-week training effect was observed in other ROIs.

#### Results of channel analysis

**Figure 4:**
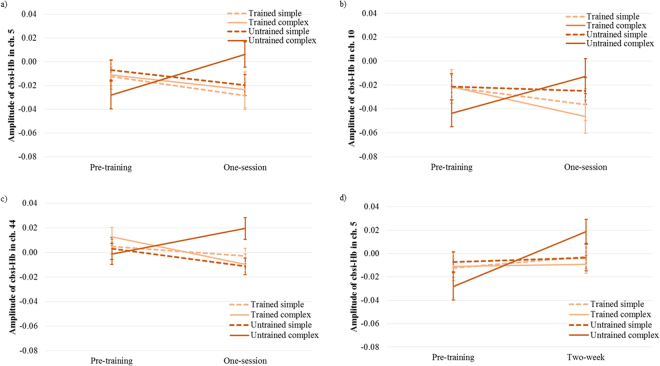
One-session training effect on brain activation changes: (**a**) in channel 5 (left AG), (**b**) channel 10 (left AG), and (**c**) channel 44 (right SPL and IPS). (**d**) Two-week training effect on brain activation changes in channel 5 (left AG). Error bars reflect SEs. Ch.: channel.

*One-session training*. The rmANOVAs on parietal channels were conducted separately. With respect to one-session training at the left AG, a significant interaction of measurement time × training was observed in channel 5 [*F*(1,19) = 4.61, *p* = 0.045, η^2^ = 0.20] (cf. Fig. 4a) and channel 10 [*F*(1,19) = 5.74, *p* = 0.027, η^2^ = 0.23] (cf. Fig. 4b). In order to explore training effects for simple and complex problems, 2 × 2 rmANOVAs were conducted in simple and complex conditions separately. No significant training effect in simple conditions was found. In complex conditions, a significant interaction of measurement time × training showed a decreased activation in the trained condition and an increased activation in the untrained condition in channel 5 [*F*(1,19) = 6.63, *p* = 0.019, η^2^ = 0.26] and channel 10 [*F*(1,19) = 8.61, *p* = 0.009, η^2^ = 0.31]. Further analysis revealed only significantly increased activation in the untrained complex condition after training in channel 5 [*t*(19) = 2.25, *p* = 0.037, *d* = 0.50] and channel 10 [*t*(19) = 2.12, *p* = 0.047, *d* = 0.47], but no significant change in the trained complex condition over time. The main effects of measurement time and training were not significant in complex conditions. Moreover, a significant interaction of measurement time × training × complexity in this region was observed in channel 10 [*F*(1,19) = 4.96, *p* = 0.038, η^2^ = 0.21]. No significant training effect was observed in channel 14. Note that the left AG is a huge region and according to the AAL atlas consists of channel 5, 10, and 14 in the present study.

In the left SPL and IPS (channel 19), a significant main effect of measurement time showed decreased activation after training [*F*(1,19) = 5.79, *p* = 0.026, η^2^ = 0.23]. There was no other significant effect in this region.

In right SPL and IPS (channel 44), a significant interaction of measurement time × training was observed [*F*(1,19) = 4.82, *p* = 0.041, η^2^ = 0.20] (cf. Fig. 4c). In order to explore training effects for simple and complex problems, 2 × 2 rmANOVAs were conducted in simple and complex conditions separately. No significant training effect was observed in simple conditions. In complex conditions, a significant interaction of measurement time × training was found [*F*(1,19) = 10.50, *p* = 0.004, η^2^ = 0.36]. Further analysis illustrated a significant difference between trained and untrained complex conditions only after training [*t*(19) = 2.52, *p* = 0.021, *d* = 0.56]. The main effects of measurement time and training were not significant. Moreover, a significant interaction of measurement time × training × complexity was observed in this region [*F*(1,19) = 6.04, *p* = 0.024, η^2^ = 0.24]. No significant training effect was observed at the right AG (channel 31, 35, and 40).

*Two-week training*. In order to define two-week training effects at the level of channels, rmANOVAs were conducted separately on parietal channels. At the left AG, a main effect of measurement time demonstrated an increased activation after training in channel 5 [*F*(1,19) = 4.41, *p* = 0.049, η^2^ = 0.19]. Furthermore, a significant interaction of measurement time × training × complexity was observed in channel 5 [*F*(1,19) = 7.02, *p* = 0.016, η^2^ = 0.27] and channel 10 [*F*(1,19) = 10.33, *p* = 0.005, η^2^ = 0.35]. In order to explore two-week training effect for simple and complex problems, two separate rmANOVAs were conducted for simple and complex multiplication. No significant result was observed in simple and complex conditions in channel 10. In channel 5, no significant training effect was found in simple conditions. In complex conditions, a significant interaction of measurement time × training showed increased activation at the left AG in untrained complex condition as compared to trained complex condition after training in channel 5 [*F*(1,19) = 8.23, *p* = 0.01, η^2^ = 0.30] (cf. Fig. 4d). Further analysis illustrated significantly increased activation only in untrained complex multiplication after training [*t*(19) = 3.74, *p* = 0.001, *d* = 0.84], but no significant change in the trained complex condition over time. Moreover, significantly greater activation was observed in untrained complex multiplication than in trained complex multiplication in the post-training period [*t*(19) = 2.75, *p* = 0.013, *d* = 0.62]. The main effects of measurement time and training were not significant in complex conditions. No significant training effect was observed in channel 14.

In the right AG, the only significant finding was an interaction of measurement time × complexity in channel 31 [*F*(1,19) = 6.86, *p* = 0.017, η^2^ = 0.27], but no significant change in channel 35, and 40. No significant training effect was observed in bilateral SPL, IPS (channels 19, and 44).

#### Result of whole measurement area analysis

**Figure 5:**
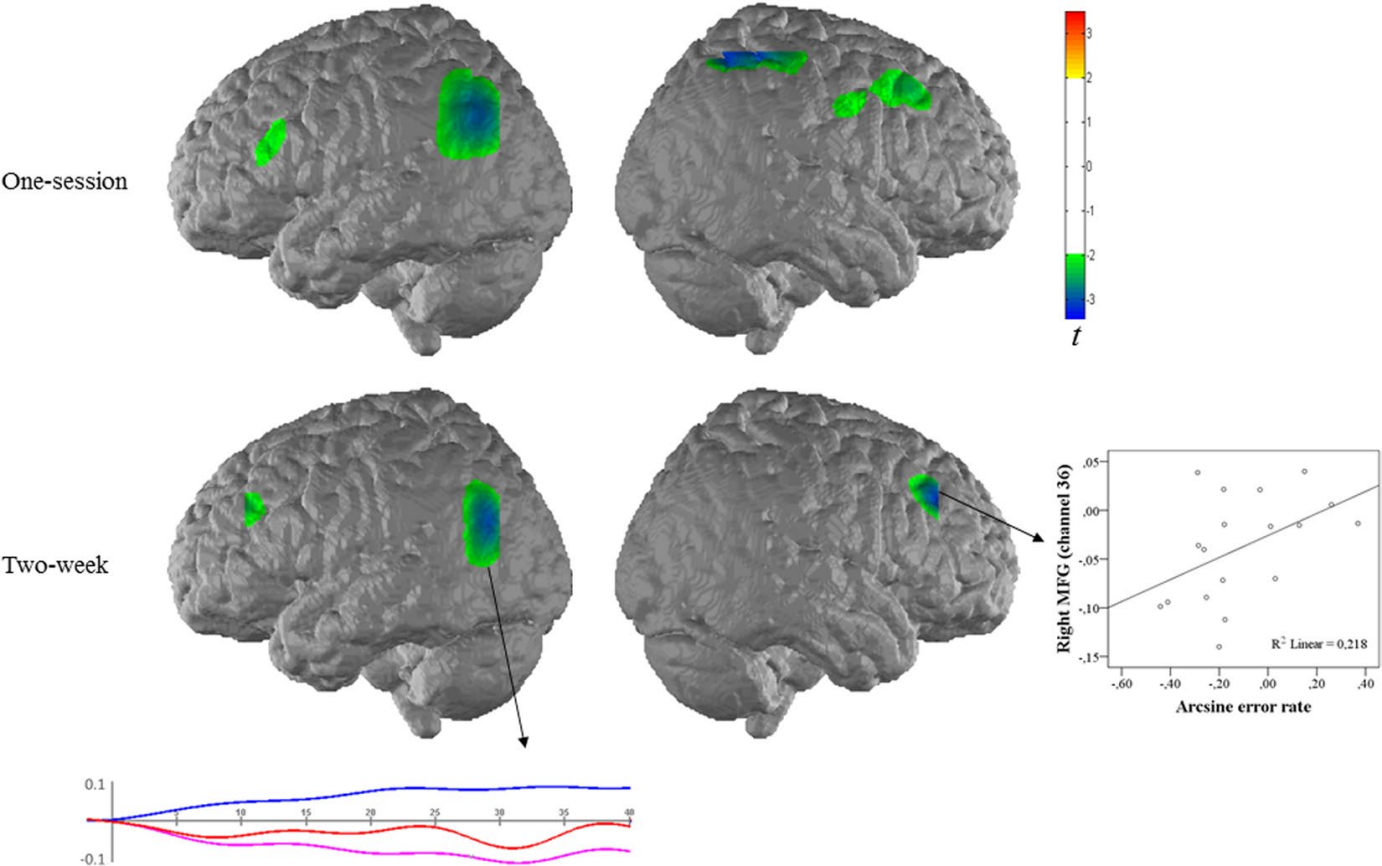
Interaction of measurement time and training in fNIRS data. The upper panel shows the one-session training effect and the lower panel shows the two-week training effect of complex multiplication on brain activation. No significant difference was observed for simple multiplication. Blue represents reduced activation, and green represents non-significant reduction of activation. The right plot shows significant correlation between error rate and activation in the right MFG for the effect of two-week training. The below plot depicts an exemplary time course of the fNIRS signal for the two-week contrast of contrast at the left AG (channel 5), which revealed a significant decrease of activation. The block average of b-values for O_2_Hb (red), HHb (blue) and CBSI-corrected signal (pink) are given.

*One-session training*. In order to assess the one-session training effect over the whole measurement area, all channels were taken into account. No significant brain activation change for simple multiplication problems was found. In complex conditions, multiplication training led to a significantly decreased activation at the left AG (channel 10), and in the right SPL and IPS (channel 44) [*ts*(19) < −2.75, D/AP corrected *ps* < 0.05, *ds* > 0.62] (cf. Fig. 5). This decrease showed less activation of the left AG, the right SPL, and IPS in trained than untrained complex multiplication after one session of training.

*Two-week training*. No training effect was observed after two weeks of training on simple multiplication problems. In complex conditions, multiplication training led to a significantly decreased activation at the left AG (channel 5) (cf. Fig. 5), and in the right MFG (channel 36) [*ts*(19) < −2.93, D/AP corrected *ps* < 0.05, *ds* > 0.66] (see Fig. 5). In other words, activation of the left AG and the right MFG decreased after two weeks of training in trained compared to untrained complex multiplication problems.

### EEG

#### Results of ROI analysis

*One-session training*. The rmANOVA on ROIs did not show significant effects of one-session training either in the theta or the alpha band.

*Two-week training*. With respect to two-week training, the rmANOVA on ROIs revealed a significant main effect of complexity in the left occipito-parietal and in middle fronto-central regions in the theta band [Fs(1,19) > 4.7, ps < 0.042, η^2^ > 0.19], showing greater theta ERS in complex than in simple conditions^27^. No other significant effect was observed in the theta frequency band. In the alpha band, two weeks of training led to a significant main effect of measurement time in occipito-parietal regions bilaterally [*Fs*(1,19) > 4.7, *ps* < 0.042, η^2^ > 0.19], which demonstrated increased alpha ERD after the training. No other significant effect was observed in the alpha frequency band.

#### Result of whole measurement area analysis

**Figure 6:**
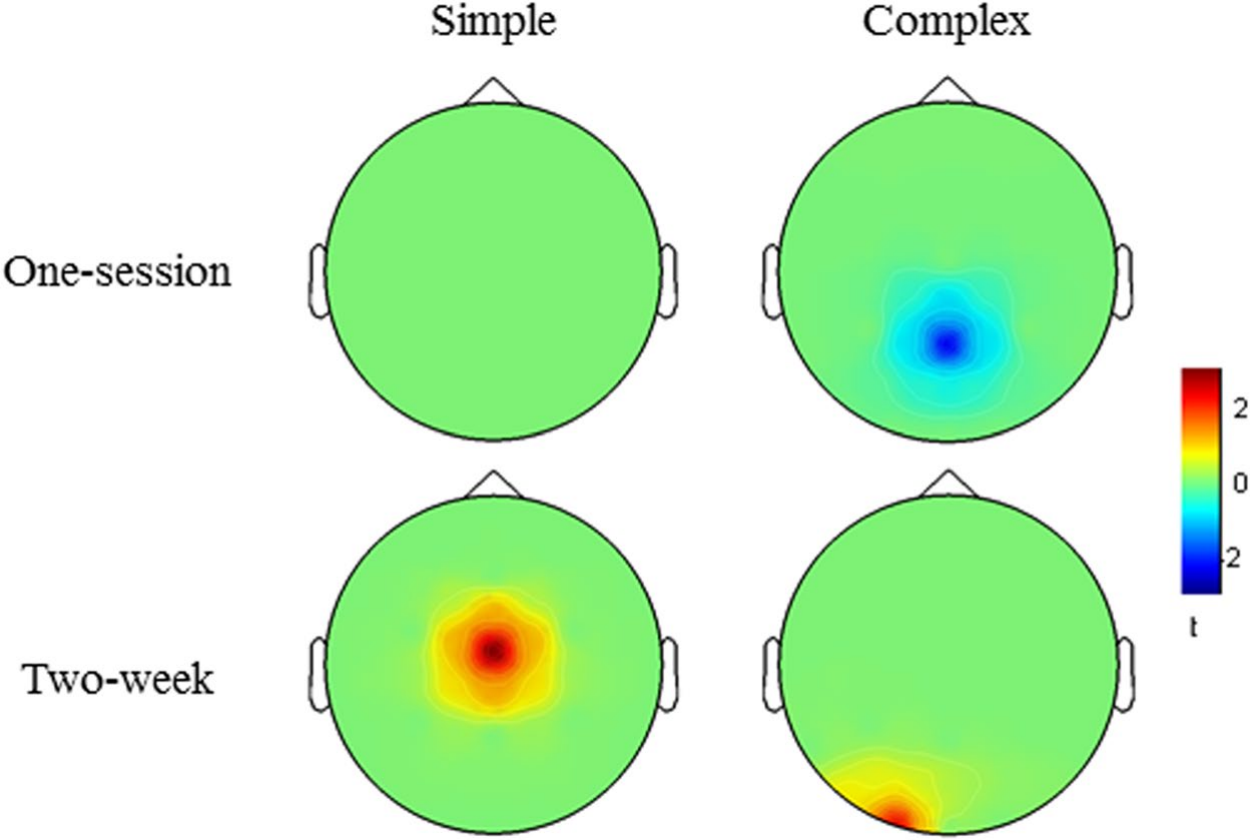
Interaction of measurement time and training in EEG data. The upper panel shows the one-session training effect and the lower panel shows the two-week training effect of simple and complex multiplication on the alpha ERD in children. No significant difference was observed in theta ERS. The red represents reduced alpha ERD and blue represents increased alpha ERD.

*One-session training*. In order to see plausible one-session training effects in the whole measurement area, similar to the fNIRS data analysis, all electrodes were taken into account. No significant training change was found for simple multiplication problems. In complex multiplication training, significantly greater alpha ERD over parietal areas (Pz) was observed in the contrast between trained and untrained problems [*t*(19) = −2.36, *p* < 0.05, *d* = 0.53] (see Fig. 6), which stems mostly from the post-training comparison (see SI, Fig. S5). Children’s behavioral performance in these two conditions was directly compared in the post-training interval: they showed significantly better performance in trained complex than in untrained multiplication problems [*t*(19) = 3.37, *p* = 0.003, *d* = 0.75]. No significant difference was observed in the theta frequency band in complex multiplication.

*Two-week training*. Two weeks of training led to significantly decreased alpha ERD at the central site (Cz) in the contrast between trained and untrained simple multiplication problems [*t*(19) = 3.11, *p* < 0.05, *d* = 0.70]. In the complex multiplication condition, significantly decreased alpha ERD was observed at the left occipital site (O1) in the contrast between trained and untrained problems [*t*(19) = 2.44, *p* < 0.05, *d* = 0.55] (cf. Fig. 6). No significant difference was observed in the theta frequency band, either in simple or in complex multiplication.

### Other neuropsychological and arithmetic tests

Regarding the transfer of multiplication learning to other arithmetic operations, a significant main effect of measurement time showed that children responded correctly to more arithmetic problems after the training [*F*(1,19) = 6.12, *p* = 0.02, η^2^ = 0.24]. A significant main effect of operation revealed that children were more competent with some basic arithmetic operations than others [*F*(1,19) = 30.89, *p* < 0.001, η^2^ = 0.62]. Ranked in order of decreasing performance, these operations were: addition > subtraction > multiplication and division [*ts*(19) > 3.03, *ps* < 0.007, *ds* > 0.68]. The interaction of measurement time × operation was not significant.

The result of a comparison between strategies used before and after training revealed that after training, children used significantly more retrieval strategies [*M*_pre-training_ = 18.75%, M _post-training_ = 37.50%, *t*(19) = 3.87, *p* = 0.001, *d* = 0.87], and fewer procedural strategies [*M*_pre-training_ = 75.63%, M _post-training_ = 60.00%, *t*(19) = −2.70, *p* = 0.014, *d* = 0.60], compared to pre-training. Furthermore, significant correlations between behavioral performance and neuropsychological tests, particularly verbal working memory, were reported in SI, Table S5.

### Brain-behavioral correlation

In order to explore the relationship between brain and behavioral findings, correlation analyses were conducted between RTs, error rates, and inverse efficiency scores with significant fNIRS and EEG findings. The analyses were conducted separately for one-session and two-week training effects. For both brain and behavioral findings, the interactions were inserted into the correlation matrices (e.g., [trained complex _post-training_ − untrained complex _post-training_] − [trained complex _pre-training_ − untrained complex _pre-training_]). Regarding the one-session training effect, no significant correlation was observed between the brain and behavioral findings, *rs*(16) < 0.30, *ps* > 0.23. Based on the Cook’s distance, two participants were found to influence and bias the correlation^70^; therefore, they were excluded from the correlation analysis of one-session training. With respect to the two-week training effect, a significant correlation between error rates and the right MFG (channel 36) was observed, *r*(18) = 0.53, *p* = 0.017 (cf. Table 1), showing that children who improved more relied less on activation of the right MFG (cf. Fig. 5). There was no significant correlation between other brain and behavioral findings in simple or complex multiplication tasks after two weeks of training.

**Table 1:** Brain-behavioral correlations for the effect of two-week training of complex multiplication.

	Brain area (channel)	RT	Error rate	Inverse efficiency
Two-week training	left AG (channel 5)	0.07	−0.32	−0.11
	right MFG (channel 36)	0.05	0.53*	0.33
	alpha ERD over left occipital (O1)	−0.14	0.01	−0.07

Note: *p < 0.05, two-tailed.

## Discussion

In the current study, a group of typically developing children received training on simple and complex multiplication problems, which led to improved performance after two weeks of training but not after a single session of training. Nevertheless, even after one session, brain activation changes were observed in the parietal lobe. After two weeks, we found a behavioral improvement associated with a strategy shift from slow, effortful, procedural to fast, compacted, procedural and retrieval strategies. This improvement was also accompanied by reduced activation of the fronto-parietal network and alpha ERD.

### One-session training effect

In the absence of any significant behavioral improvement, fNIRS findings showed reduced activation at the right IPS and SPL after one training session in the trained complex condition. The findings in the right IPS and SPL are in line with a longitudinal (non-learning) study in children, which reported that one year of academic education led to reduced activation of the right parietal-occipital cortex during addition problem-solving^22^. This result is also supported by studies in adults^2^. Decreased activation of the right IPS, which is related to quantity-based processing, indicates that after the training, children needed less “manipulation” of the numerical magnitudes. Moreover, in the present study, a production paradigm was used, and the problems required an exact calculation, so other plausible strategies such as approximation could not be used^3^. Therefore, it is reasonable to suppose that the decreased activation of the right IPS was related to changes in the process of exact calculation^9^. Additionally, we observed reduced activation of the right SPL, which supports domain-general attention processes related to numerical processing. This finding shows that arithmetic learning lessens demands on domain-general supporting areas in children, as in adults. An unexpected decrease in activation of the left AG was detected during trained, complex multiplication problems compared to untrained, complex problems, after a single session of training. Note that this reduction comes from the interaction between measurement time and training. Further exploration of this interaction revealed no significant change in the left AG after training, in the trained complex condition, but an increased activation in the untrained, complex condition (see Fig. 4). Bayesian analysis renders this as positive evidence^71^ for the absence of a change in activation in the trained condition (posterior probability of 0.81 for channel 5 and 0.73 for channel 10). This finding is supported by previous studies in children^22,23^, but is contradictory to multiplication training studies in adults, which reported increased activation of the left AG after short-term training^2,6^. The left AG activation in children will be further discussed below.

Regarding EEG, an increased alpha ERD as a result of one session of complex multiplication training was unexpected. One possible explanation is that alpha ERD may be sensitive to visual attention processes^72^. Because performance was better and response times were faster during trained complex problems than untrained complex problems in post-training measurement time (see SI, Figs S2 and S3), more problems were presented in the trained complex condition, resulting in more visual processing that may have induced alpha ERD. In simple multiplication, no significant difference was observed, which might be due to insufficient training; children in grade 5 are usually already proficient in solving simple multiplication problems, and more repetitions than just a single session would be needed to improve their performance. In fact, because of a potential ceiling effect, even more training sessions might not elicit improvement at this stage of development.

### Brain activation changes in children after two weeks of training

The two-week training data illustrated that children became more efficient in the trained than the untrained multiplication problems, meaning that they provided faster responses with fewer errors. With respect to the trained simple condition, no significant change was observed in the fNIRS data. However, decreased alpha ERD was found in EEG data for both simple and complex trained multiplication problems^8^. This decrease suggests an enhancement in fast, compacted procedural and retrieval processing in both trained conditions. This finding is in line with previous studies in which working memory training^69^ and multiplication training^8^ led to decreased alpha ERD, or reduced cortical activation. Alpha ERD represents a reduction of localized amplitudes, associated with the increased excitability of cortical regions^29^ and increased reliance on retrieval strategies. This cortical excitability reflects a rise in information processing. Therefore, decreased alpha ERD in both trained conditions in the present study can be interpreted as a reduction in cortical effort. It is important to note, however, that although EEG results corresponded to our *a priori* hypothesis, they did not survive correction for multiple comparisons, and therefore need to be considered with caution. Nevertheless, we believe that because of their convergence with the fNIRS data, the EEG data contribute to the accurate interpretation of our findings.

In the trained complex condition, fNIRS findings showed reduced activation at the right MFG after training, in comparison with the untrained complex condition. It has been shown that learning converts general-purpose or domain-general processing requirements to more domain-specific requirements, which is indicated by reductions in activity in several brain regions^73^. The reduced activation in the MFG is further supported by the reduction in alpha ERD after two weeks of training, which suggests a decrease in the demands placed by general cognitive processes, and an increased reliance on retrieval strategies, in line with an oscillatory EEG study of arithmetic learning in adults^8^. The reduced activation in the MFG is also in line with previous fMRI studies of multiplication training in adults, which have reported a decreased activation within the frontal lobe^2^. In the present study, the right MFG and the alpha band, both involved in executive control and working memory^27^, showed reduced activation after the training. This indicates faster calculation processes after complex multiplication training that do not depend as much on sequential cognitive processes as before training^24^. Interestingly, reduced activation of the right MFG was associated with the error rate, showing that children who improved the most with the training subsequently relied less on domain-general cognitive processing to solve the tasks.

A decrease in activation at the left AG was detected during the completion of trained, complex multiplication problems, compared to untrained problems, after two weeks of training. This reduction is formally described by the interaction between measurement time and training, as in the one-session training results. Further exploration of this interaction revealed no significant change in the left AG after training, in the trained, complex condition, but an increase in activation in the untrained, complex condition. Bayesian analysis established the lack of activation change in the left AG as positive evidence^71^ for the absence of change in the trained condition (posterior probability of 0.81 for channel 5). This finding is supported by previous studies in children^22,23^, but contradicts multiplication training studies in adults, which have reported increased activation of the left AG after training^2^. Note that a recent study in adults^26^ reported no activation changes in the left AG in the comparison between complex multiplication problems solved before versus after training. However, while the post-training comparison between trained and untrained complex multiplication problems in adults shows an activation increase in the left AG, we observed an activation decrease in the left AG in children (cf. SI, Fig. S4). It seems that although a shift from procedural effortful to retrieval memory-based strategies is represented in adults as a shift from the frontal to the parietal lobe, and then within the parietal lobe to the left AG, the same is not necessarily true for children^23^. This difference might be due to more stable neural substrates of arithmetic processes in adults compared to children^19,22^. Furthermore, this strategy shift is not represented by similar brain activation changes from childhood into adulthood^22^. It should be noted that even for adults, different brain areas, and not only the left AG, are involved in retrieval processes after multiplication training^4,11,26^. Furthermore, several studies have shown an unspecific role of the left AG activation in arithmetic learning^5,74,75^.

We observed an unexpected increase in activation of the left AG in untrained complex multiplication post-training compared to the pre-training session. Note that the only adults’ study which has investigated the contrast between post-training and pre-training^26^ reported no activation changes. Therefore, this finding for the untrained condition in the present study also conflicts with previous data from adults. This increased activation may reflect improved performance (i.e., faster responses; see SI, Fig. S2) in untrained complex multiplication via training, which might be due to increased recruitment of domain-general regions. This is different from trained complex multiplication, which showed less brain activity with improved performance via training, probably because effort-saving retrieval processes are recruited here. It seems that short-term arithmetic training leads to a restricted generalization to the other problems of the same operation. This restricted generalization means that training leads to an improvement in both trained and untrained problem-solving, but this improvement is much stronger in the case of trained problems. In the current study, this restricted generalization was detected in the response times after two weeks of training (see SI, Fig. S2). Children responded faster to not only both trained sets but also to both untrained sets. This restricted generalization has been already shown in adults^7^, and also depends on the training method^4^. In sum, this shows that better performance might be subserved by different neurocognitive mechanisms: (i) efficient recruitment of specific areas associated with strategy change (e.g., procedural to retrieval processes) when the particular items have been trained, or (ii) recruitment of more brain areas associated with domain-general processes within the same (procedural) strategy, when the particular items have not been trained, but the outcome of the procedural strategy itself is improved. We conclude that the AG might have an intermediate role during development, with a nonlinear relation (over age and development) between the AG activation increase/decrease and arithmetic learning. However, this assumption needs to be tested in larger future studies that use the same learning paradigm over a wide range of age groups.

### Transfer effects

With respect to transfer effects, a generally improved performance in all basic arithmetic operations was found after multiplication training in children, which was not specific to one operation. However, even though the time interval between pre- and post-training measurement was short (two weeks) and children in grade 5 do not receive direct training in basic arithmetic, the absence of a control group makes it difficult to interpret this improvement as the result of multiplication training.

### Possible methodological and analytical differences

While the different findings of arithmetic training between adults and children can be explained by the above neurocognitive accounts focusing on different brain-behavior relations between children and adults, there are some alternative methodological explanations that should be mentioned and possibly tested in future studies. First, while most training studies have used verification paradigms to reduce movement artifacts in the MRI scanner, the present study applied a written production paradigm. This means that children calculated almost every single trial without using any shortcut strategies. In other studies, the presence of visible response options might promote the use of retrieval strategies. Secondly, we used blocked conditions and a self-paced design (as in most cognitive and educational settings). The self-paced design ensured that children continuously performed the tasks, without larger resting times between items in easier (faster) problem sets. This was accomplished by presenting a higher number of trials in the easier conditions, allowing children move to the next item as soon as they responded.

Note that every design has its strengths and weaknesses: (i) The advantage of the self-paced block design is that the time spent on the task is equivalent for simpler or more complex trials. The typical convolution with the HRF should apply similarly to both types of trials, because the participant is constantly performing the task. However, this comes with the trade-off that the participant do more trials (but not more time on one task). Therefore, additional processes like switching from trial to trial or more response preparation when more responses are needed in block design, are then confounded. (ii) In a fixed-pace block design, while the number of trials and the length of the signal remain identical, these lengths do not strictly represent mental calculation if participants solve problems before the response window expires. (iii) A self-paced event-related design has the advantage of an identical number of trials per condition and participant. However, because of huge inter-individual differences between children, the lengths of signals differ dramatically. Usually the BOLD response in trials that last longer is more expanded over time. Therefore, it is not clear whether more activation as indicated by the HRF is actually due to greater difficulty (i.e., greater intensity of brain activation) or just to a greater length of time needed to process the task, and therefore a longer duration when the brain is working on the task. It might be of interest to compare brain activation changes in different designs in future studies.

## Conclusion

The present study shows that performance improvement via arithmetic learning in children is accompanied by brain activation changes, as measured by simultaneous fNIRS-EEG. However, these changes differed from those induced by arithmetic training in adults. While studies in adults have reported a shift from procedural to retrieval strategies, marked by decreased activation in frontal gyri, the IPS, and the SPL, and increased activation of the left AG, the present training study in children revealed generally decreased brain activation. This difference might be because of an extended brain network for arithmetic processing in children compared to adults^15,19^. We interpret these differences in brain activation changes as an effect of age, suggesting that the strategy shift in children has a different neural pattern than in adults, although some alternative methodological accounts should be addressed in future studies. Moreover, activation change in the left AG depends on the contrast as well, which needs to be taken into account for interpretation of the findings. Independent of the explanations for our results, one take-home message is clear: previous findings from experimental neurocognitive studies in adults cannot be simply generalized to children’s arithmetic learning, particularly in an ecologically valid setting resembling arithmetic performance in schools. Therefore, in a more general conclusion, we argue that this study is an example of the Educational Neuroscience Approach, studying educational contents and settings with neuroscientific methods. Furthermore, it is necessary to understand (neurocognitive) development and learning in children – experimental neurocognitive studies in adults alone will not be sufficient.

## Electronic supplementary material

Supplementary information

## References

[1] Butterworth B, Varma S, Laurillard D (2011). Dyscalculia: from brain to education. science.

[2] Zamarian L, Ischebeck A, Delazer M (2009). Neuroscience of learning arithmetic—evidence from brain imaging studies. Neuroscience & Biobehavioral Reviews.

[3] Delazer M (2003). Learning complex arithmetic—an fMRI study. Cognitive Brain Research.

[4] Delazer M (2005). Learning by strategies and learning by drill—evidence from an fMRI study. Neuroimage.

[5] Ischebeck A (2006). How specifically do we learn? Imaging the learning of multiplication and subtraction. Neuroimage.

[6] Ischebeck A, Zamarian L, Egger K, Schocke M, Delazer M (2007). Imaging early practice effects in arithmetic. Neuroimage.

[7] Ischebeck A, Zamarian L, Schocke M, Delazer M (2009). Flexible transfer of knowledge in mental arithmetic—An fMRI study. Neuroimage.

[8] Grabner, R. H. & De Smedt, B. Oscillatory EEG correlates of arithmetic strategies: a training study. *Frontiers in psychology* **3** (2012). 10.3389/fpsyg.2012.00428 PMC3498901 23162495

[9] Dehaene S, Piazza M, Pinel P, Cohen L (2003). Three parietal circuits for number processing. Cognitive neuropsychology.

[10] Klein E, Moeller K, Glauche V, Weiller C, Willmes K (2013). Processing pathways in mental arithmetic—evidence from probabilistic fiber tracking. PloS one.

[11] Klein E (2016). Considering structural connectivity in the triple code model of numerical cognition: differential connectivity for magnitude processing and arithmetic facts. Brain Structure and Function.

[12] Grabner RH (2007). Individual differences in mathematical competence predict parietal brain activation during mental calculation. Neuroimage.

[13] Amalric, M. & Dehaene, S. Origins of the brain networks for advanced mathematics in expert mathematicians. *Proceedings of the National Academy of Sciences*, 201603205 (2016). 10.1073/pnas.1603205113 PMC4983814 27071124

[14] Peters, L. & De Smedt, B. Arithmetic in the developing brain: A review of brain imaging studies. *Developmental Cognitive Neuroscience* (2017). 10.1016/j.dcn.2017.05.002 PMC6969129 28566139

[15] Arsalidou, M., Pawliw-Levac, M., Sadeghi, M. & Pascual-Leone, J. Brain areas needed for numbers and calculations in children: Meta-analyses of fMRI studies. *Developmental Cognitive Neuroscience* (2017). 10.1016/j.dcn.2017.08.002 PMC6969084 28844728

[16] Geary, D. C. *Children’s mathematical development: Research and practical applications*. (American Psychological Association, 1994).

[17] Siegler, R. S. *Emerging minds: The process of change in children’s thinking*. (Oxford University Press, 1996).

[18] Dimitriadis SI, Laskaris NA, Micheloyannis S (2015). Transition dynamics of EEG-based network microstates during mental arithmetic and resting wakefulness reflects task-related modulations and developmental changes. Cogn Neurodynamics.

[19] Micheloyannis S (2009). The influence of ageing on complex brain networks: a graph theoretical analysis. Human brain mapping.

[20] Rocha FT, Rocha AF, Massad E, Menezes R (2005). Brain mappings of the arithmetic processing in children and adults. Cognitive Brain Research.

[21] Rivera SM, Reiss A, Eckert MA, Menon V (2005). Developmental changes in mental arithmetic: evidence for increased functional specialization in the left inferior parietal cortex. Cerebral Cortex.

[22] Qin S (2014). Hippocampal-neocortical functional reorganization underlies children’s cognitive development. Nature neuroscience.

[23] Supekar K (2013). Neural predictors of individual differences in response to math tutoring in primary-grade school children. Proceedings of the National Academy of Sciences.

[24] Prado J, Mutreja R, Booth JR (2014). Developmental dissociation in the neural responses to simple multiplication and subtraction problems. Developmental science.

[25] De Smedt B, Holloway ID, Ansari D (2011). Effects of problem size and arithmetic operation on brain activation during calculation in children with varying levels of arithmetical fluency. Neuroimage.

[26] Bloechle, J. *et al*. Fact learning in complex arithmetic—the role of the angular gyrus revisited. *Human Brain Mapping* (2016). 10.1002/hbm.23226 PMC6867278 27130734

[27] Soltanlou, M. *et al*. Increased arithmetic complexity is associated with domain-general but not domain-specific magnitude processing in children: A simultaneous fNIRS-EEG study. *Cognitive, Affective, & Behavioral Neuroscience*, 1–13 (2017). 10.3758/s13415-017-0508-x 28474293

[28] Hinault, T. & Lemaire, P. What does EEG tell us about arithmetic strategies? A review. *International Journal of Psychophysiology* (2016). 10.1016/j.ijpsycho.2016.05.006 27220781

[29] Pfurtscheller G (2001). Functional brain imaging based on ERD/ERS. Vision research.

[30] Antonenko P, Paas F, Grabner R, van Gog T (2010). Using electroencephalography to measure cognitive load. Educational Psychology Review.

[31] Grabner RH, De Smedt B (2011). Neurophysiological evidence for the validity of verbal strategy reports in mental arithmetic. Biological psychology.

[32] Harmony Ta (1999). Do specific EEG frequencies indicate different processes during mental calculation?. Neuroscience letters.

[33] Micheloyannis S, Sakkalis V, Vourkas M, Stam CJ, Simos PG (2005). Neural networks involved in mathematical thinking: evidence from linear and non-linear analysis of electroencephalographic activity. Neuroscience letters.

[34] Moeller K, Wood G, Doppelmayr M, Nuerk H-C (2010). Oscillatory EEG correlates of an implicit activation of multiplication facts in the number bisection task. Brain research.

[35] Fendrich, D. W., Healy, A. F. & Bourne, L. E. Jr. In *Cognitive Psychology Applied: A Symposium at the 22nd International Congress of Applied Psychology*. 111 (Psychology Press).

[36] Menon V, Rivera S, White C, Glover G, Reiss A (2000). Dissociating prefrontal and parietal cortex activation during arithmetic processing. Neuroimage.

[37] Huber, S., Fischer, U., Moeller, K. & Nuerk, H.-C. On the interrelation of multiplication and division in secondary school children. *Frontiers in psychology* **4** (2013). 10.3389/fpsyg.2013.00740 PMC3794290 24133476

[38] Rorden C, Brett M (2000). Stereotaxic display of brain lesions. Behavioural neurology.

[39] Singh AK, Okamoto M, Dan H, Jurcak V, Dan I (2005). Spatial registration of multichannel multi-subject fNIRS data to MNI space without MRI. Neuroimage.

[40] Tsuzuki D (2007). Virtual spatial registration of stand-alone fNIRS data to MNI space. Neuroimage.

[41] Tzourio-Mazoyer N (2002). Automated anatomical labeling of activations in SPM using a macroscopic anatomical parcellation of the MNI MRI single-subject brain. Neuroimage.

[42] Jasper HH (1958). The ten twenty electrode system of the international federation. Electroencephalography and clinical neurophysiology.

[43] Oostenveld R, Praamstra P (2001). The five percent electrode system for high-resolution EEG and ERP measurements. Clinical neurophysiology.

[44] Petermann, F., Petermann, U. & Wechsler, D. *Hamburg-Wechsler-Intelligenztest für Kinder-IV: HAWIK-IV* (Huber, 2007).

[45] Alloway TP, Gathercole SE, Pickering SJ (2006). Verbal and Visuospatial Short‐Term and Working Memory in Children: Are They Separable?. Child development.

[46] Corsi, P. M. *Human memory and the medial temporal region of the brain*, ProQuest Information & Learning, (1973).

[47] Soltanlou, M., Pixner, S. & Nuerk, H.-C. Contribution of working memory in multiplication fact network in children may shift from verbal to visuo-spatial: a longitudinal investigation. *Frontiers in psychology* **6** (2015). 10.3389/fpsyg.2015.01062 PMC4512035 26257701

[48] Jung, S. *et al*. In *Advances in Computers and Technology for Education–Proceedings of the 11th International Conference on Educational Technologies*. 13-22.

[49] Jung, S. *et al*. Die TUebinger LernPlattform zum Erwerb numerischer und orthografischer Kompetenzen (TULPE): individualisierte Förderung durch adaptive Lernspiele. *Lernen und Lernstörungen* **5** (2016).

[50] Roesch, S. *et al*. Training arithmetic and orthography on a web-based and socially-interactive learning platform. *International Journal of Education and Information Technologies* (2016).

[51] Butterworth, B. *Dyscalculia screener*. (nferNelson Pub., 2003).

[52] Winer, B. J., Brown, D. R. & Michels, K. M. *Statistical principles in experimental design*. Vol. 2 (McGraw-Hill New York, 1971).

[53] Lakens, D. Calculating and reporting effect sizes to facilitate cumulative science: a practical primer for t-tests and ANOVAs. *Frontiers in psychology* **4** (2013). 10.3389/fpsyg.2013.00863 PMC3840331 24324449

[54] Haeussinger FB (2014). Reconstructing functional near-infrared spectroscopy (fNIRS) signals impaired by extra-cranial confounds: an easy-to-use filter method. NeuroImage.

[55] Sasai S, Homae F, Watanabe H, Taga G (2011). Frequency-specific functional connectivity in the brain during resting state revealed by NIRS. Neuroimage.

[56] Tong Y (2010). Time lag dependent multimodal processing of concurrent fMRI and near-infrared spectroscopy (NIRS) data suggests a global circulatory origin for low-frequency oscillation signals in human brain. Neuroimage.

[57] Zuo X-N (2010). The oscillating brain: complex and reliable. Neuroimage.

[58] Plichta MM, Heinzel S, Ehlis AC, Pauli P, Fallgatter AJ (2007). Model-based analysis of rapid event-related functional near-infrared spectroscopy (NIRS) data: A parametric validation study. Neuroimage.

[59] Scholkmann F (2014). A review on continuous wave functional near-infrared spectroscopy and imaging instrumentation and methodology. Neuroimage.

[60] Cui X, Bray S, Reiss AL (2010). Functional near infrared spectroscopy (NIRS) signal improvement based on negative correlation between oxygenated and deoxygenated hemoglobin dynamics. Neuroimage.

[61] Brigadoi S (2014). Motion artifacts in functional near-infrared spectroscopy: a comparison of motion correction techniques applied to real cognitive data. Neuroimage.

[62] Sankoh AJ, Huque MF, Dubey SD (1997). Some comments on frequently used multiple endpoint adjustment methods in clinical trials. Statistics in medicine.

[63] Tadel F, Baillet S, Mosher JC, Pantazis D, Leahy RM (2011). Brainstorm: a user-friendly application for MEG/EEG analysis. Computational intelligence and neuroscience.

[64] Tesche C (1995). Signal-space projections of MEG data characterize both distributed and well-localized neuronal sources. Electroencephalography and clinical neurophysiology.

[65] Pfurtscheller G, Da Silva FL (1999). Event-related EEG/MEG synchronization and desynchronization: basic principles. Clinical neurophysiology.

[66] Pfurtscheller G, Aranibar A (1977). Event-related cortical desynchronization detected by power measurements of scalp EEG. Electroencephalography and clinical neurophysiology.

[67] Klimesch W (1999). EEG alpha and theta oscillations reflect cognitive and memory performance: a review and analysis. Brain research reviews.

[68] Nunez, P. L. & Cutillo, B. A. *Neocortical dynamics and human EEG rhythms*. (Oxford University Press, USA, 1995).

[69] Gevins A, Smith ME, McEvoy L, Yu D (1997). High-resolution EEG mapping of cortical activation related to working memory: effects of task difficulty, type of processing, and practice. Cerebral cortex.

[70] Field, A. *Discovering statistics using IBM SPSS statistics*. (Sage, 2013).

[71] Masson ME (2011). A tutorial on a practical Bayesian alternative to null-hypothesis significance testing. Behavior research methods.

[72] Klimesch W, Sauseng P, Hanslmayr S (2007). EEG alpha oscillations: the inhibition–timing hypothesis. Brain research reviews.

[73] Poldrack RA (2000). Imaging brain plasticity: conceptual and methodological issues—a theoretical review. Neuroimage.

[74] Grabner RH (2009). Fact learning in complex arithmetic and figural‐spatial tasks: The role of the angular gyrus and its relation to mathematical competence. Human Brain Mapping.

[75] Simon O, Mangin J-F, Cohen L, Le Bihan D, Dehaene S (2002). Topographical layout of hand, eye, calculation, and language-related areas in the human parietal lobe. Neuron.

