# Supplementary material for: Reduction but no shift in brain activation after arithmetic learning in children: A simultaneous fNIRS-EEG study
Source: Sci Rep. 2018 Jan 26;8:1707. doi: 10.1038/s41598-018-20007-x (PMC5786008; doi:10.1038/s41598-018-20007-x)
Supplement: Supplementary file 1 — Supplementary information [file 41598_2018_20007_MOESM1_ESM.pdf]

Supplementary information (SI) for

Reduction but no shift in brain activation after arithmetic learning in children: A simultaneous fNIRS-EEG study

Mojtaba Soltanlou, Christina Artemenko, Ann-Christine Ehlis, Stefan Huber, Andreas J. Fallgatter, Thomas Dresler, Hans-Christoph Nuerk

Material and methods

Training procedure

In the present study, 32 multiplication problems, 8 problems per condition, were used (cf. Table S1).

Table S1: List of problems in four conditions.

Trained simple	Trained complex	Untrained simple	Untrained complex
3 × 4	13 × 4	6 × 2	18 × 3
5 × 3	3 × 19	7 × 2	6 × 12
2 × 8	5 × 13	3 × 7	4 × 19
6 × 3	18 × 4	4 × 6	7 × 12
3 × 9	6 × 13	8 × 3	14 × 6
7 × 4	15 × 6	7 × 5	17 × 5
5 × 6	12 × 8	4 × 9	5 × 18
8 × 4	7 × 14	5 × 8	13 × 7

It should be noted that because the online training platform was used at home, it was not possible to fully control the training procedure. As we discussed in the paper, there were some incomplete sessions, which might influence the training effect. Therefore, based on the interval between the incomplete session and the nearest complete session preceding or following it, we considered the incomplete session part of one of these neighboring sessions. Moreover, because of some very rare technical problems in the online platform, in some sessions, the problems were presented 7 times instead of 6 times (cf. Table S2).

Table S2: Mean (and SD) of number of presented trained simple and complex problems per training session.

Session	1	2	3	4	5	6	7
Simple	49.8 (3.1)	52.3 (8.6)	50.1 (11.2)	52.9 (13.1)	51.6 (9.5)	51.2 (8.7)	50.5 (12.3)
Complex	49.9 (3.4)	52.8 (10.1)	50.8 (11.9)	53.4 (14.1)	51.2 (9.1)	51.2 (10.1)	52.0 (12.8)

For each problem, one correct solution and 11 distractors were presented. Each distractor was made based on one of the following rules: adding 1 to or subtracting 1 from the first or second operand, adding or subtracting 1, 2, 10 from the correct solution, or inversing the unit and decade of the correct solution.

Neuropsychological tests

Children's performance on IQ subtests of similarity and matrix reasoning, along with memory components (verbal STM, verbal WM, visuospatial STM, visuospatial WM), are presented in Table S3. To investigate the transfer effect of multiplication training to other operations (addition, subtraction, multiplication, division), we used two closely matched sets of all four basic arithmetic operations before and after the training. The test was a modified version of an arithmetic test[1] with two levels of complexity resulting in eight lists of problems. Children had 45s for each simple list and 60s for each complex list, and they were required to answer as many problems as possible while avoiding errors.

Table S3: Mean (and SD) of IQ subtests and memory components.

**Similarities**	**Matrix reasoning**	**Verbal STM**	**Verbal WM**	**Visuospatial STM**	**Visuospatial WM**
108.5 (11.71)	108.0 (10.44)	4.95 (0.76)	3.95 (0.89)	5.35 (0.81)	5.30 (1.13)

Note: STM: short-term memory; WM: working memory.

Analysis

FNIRS

As shown in Fig. S1 and Table S4, 4 ROIs were defined for fNIRS analysis and 6 ROIs were defined for EEG analysis.

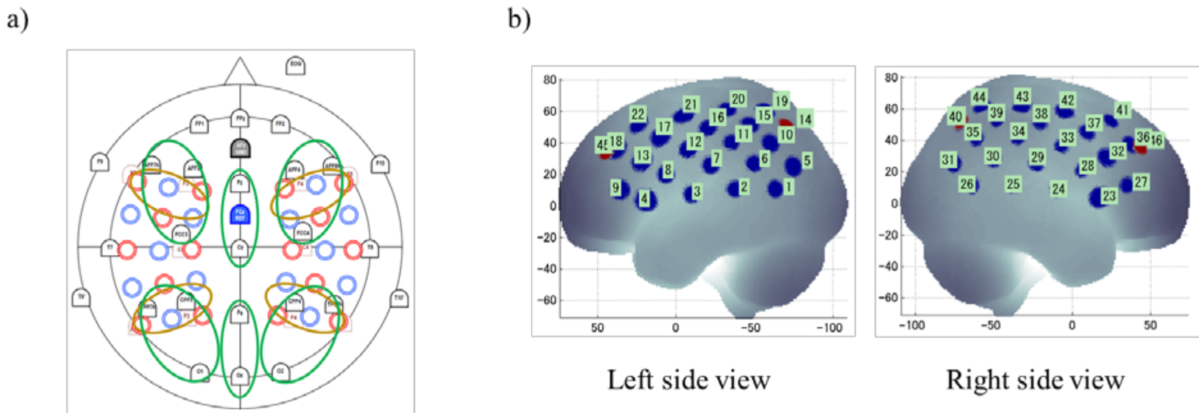

Fig. S1: a) Schematic positions of fNIRS optodes and EEG electrodes. Small red circles indicate emitters and blue ones indicate detectors in the two arrays of 3×5. Small white shapes indicate positions of the EEG electrodes. Red dotted shapes indicate the original position of some EEG electrodes according to the international 10-20 system. FNIRS ROIs are shown with brown circles, and EEG ROIs are shown with green circles. b) FNIRS channels layout and numbers. Blue circles indicate areas of channels projected on the brain surface. Red circles indicate P3, P4, F3 and F4 points projected on the brain surface (by Minako Uga).

Table S4: FNIRS and EEG ROIs.

	ROIs	Channels/Electrodes
**fNIRS**	L frontal	9, 13, 18, 22
	L parietal	5, 10, 14, 19
	R frontal	27, 32, 36, 41
	R parietal	31, 35, 40, 44
**EEG**	L frontal	AFF3, AFF7h, FCC3
	L parietal	CPP3, TPP7h, O1
	R frontal	AFF4, AFF8h, FCC4
	R parietal	CPP4, TPP8h, O2
	M frontal	Fz, Cz
	M parietal	Pz, Oz

Additionally, in order to investigate the difference between trained and untrained conditions within each measurement time (pre-training, first post-training, and second post-training), multiple paired *t*-tests were applied: trained simple versus untrained simple; trained complex versus untrained complex. The significance level was 0.05 and corrected using the Dubey/Armitage-Parmar (D/AP) method for multiple comparisons[2]. Note that the contrast of trained versus untrained in the second post-training measurement is the typical analysis that has been conducted in previous studies in adults[3].

EEG

Again similar to the fNIRS data, the contrast of trained versus untrained conditions was calculated with paired *t*-tests within each measurement time. The significant level was 0.05 uncorrected.

Results

Behavioral

RT

The analysis of median RT after one session of training revealed a significant main effect of complexity, showing that children responded faster to simple than to complex problems [$F(1,19) = 188.82$, $p < 0.001$, $\eta^2 = 0.91$]. No other significant main effect or interaction was found in the analysis of median RTs with respect to one-session training (cf. Fig. S2a).

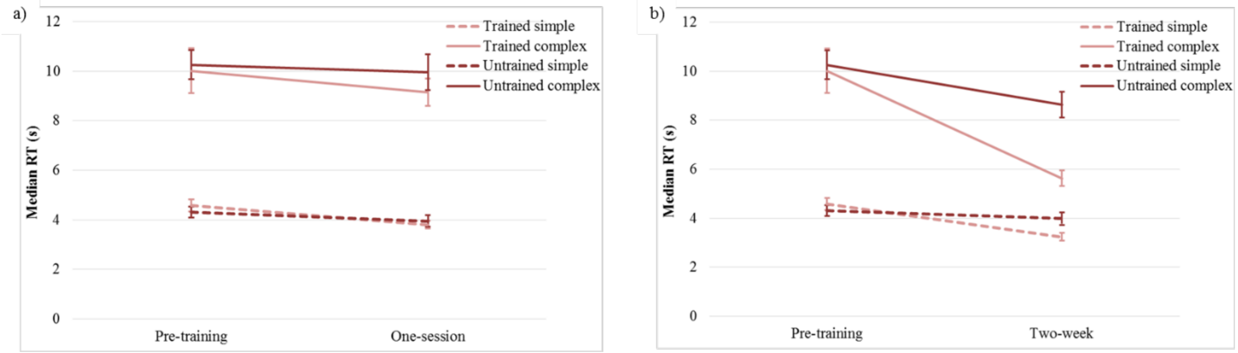

Fig. S2: a) One-session training effect and b) Two-week training effect on median RT. Error bars reflect SEs.

With respect to the median RT after two weeks of training, significant main effects of measurement time, training, and complexity were observed [$Fs(1,19) > 19.3$, $ps < 0.001$, $\eta^2 > 0.49$]. A significant main effect of measurement time indicated that children became faster after training in multiplication problem solving. A significant interaction of measurement time × training showed that training led to improved performance in trained versus untrained conditions in terms of response time [$F(1,19) = 16.14$, $p < 0.001$, $\eta^2 = 0.46$]. In order to explore training effects for simple and complex problems, two separate rmANOVAs were conducted for simple and complex multiplication. Regarding simple multiplication, a significant main effect of measurement time showed that children provided faster responses after training [$F(1,19) = 27.66$, $p < 0.001$, $\eta^2 = 0.59$]. Moreover, the significant interaction effect of measurement time × training revealed a two-week training effect in trained simple compared to untrained simple multiplication [$F(1,19) = 26.18$, $p < 0.001$, $\eta^2 = 0.58$]. Further analysis showed that children responded faster to trained simple than untrained simple problems in post-training measurement [$t(19) = 4.68$, $p < 0.001$, $d = 1.05$], while they did not differ significantly before training. The main effect of training did not reach significance in simple conditions. Regarding complex multiplication, a significant main effect of measurement time, demonstrating faster responses after training [$F(1,19) = 24.28$, $p < 0.001$, $\eta^2 = 0.56$], and a significant main effect of training [$F(1,19) = 20.70$, $p < 0.001$, $\eta^2 = 0.52$], were observed. A significant interaction effect of measurement time × training revealed that after training, children provided faster responses to trained complex compared to untrained complex problems [$F(1,19) = 9.32$, $p = 0.007$, $\eta^2 = 0.33$]. Additional analysis showed that children responded faster to trained complex than untrained complex problems in post-training measurement [$t(19) = 7.29$, $p < 0.001$, $d = 1.63$], while they did not differ significantly before training (cf. Fig. S2b).

Moreover, a significant interaction of measurement time × complexity [$F(1,19) = 16.44$, $p < 0.001$, $\eta^2 = 0.46$], and a significant interaction of training × complexity [$F(1,19) = 15.68$, $p < 0.001$, $\eta^2 = 0.45$], and a marginally significant interaction of measurement time × training × complexity [$F(1,19) = 3.67$, $p = 0.07$, $\eta^2 = 0.16$] were observed (see Fig. S2b).

Error rate

Regarding the error rate after one session of training, a significant main effect of complexity demonstrated that children responded more accurately to simple compared to complex problems [$F(1,19) = 105.23$, $p < 0.001$, $\eta^2 = 0.85$]. Moreover, a significant interaction of training × complexity was observed [$F(1,19) = 7.89$, $p = 0.011$, $\eta^2 = 0.29$]. No other significant effect was found in analysis of error rate after one session of training (see Fig. S3a).

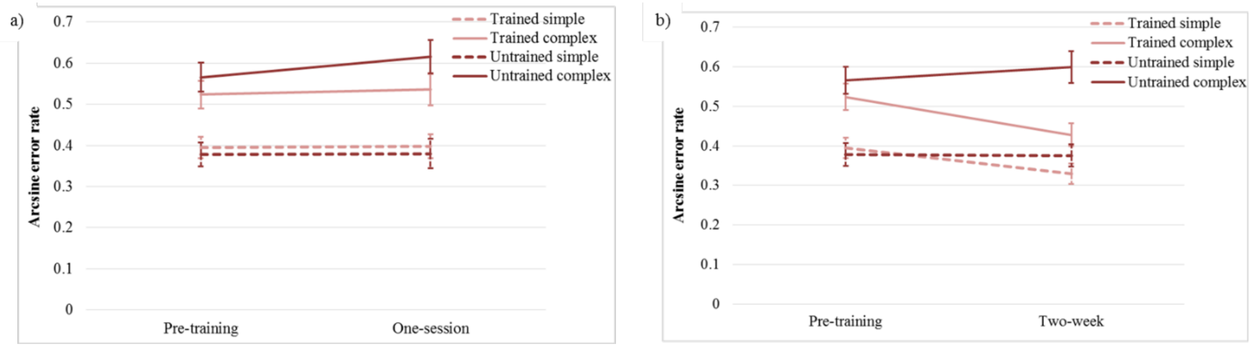

Fig. S3: a) One-session training effect and b) Two-week training effect on arcsine error rate. Error bars reflect SEs.

With respect to two-week training, a similar rmANOVA over the error rate displayed a significant main effect of training [$F(1,19) = 9.83$, $p = 0.005$, $\eta^2 = 0.34$], and complexity [$F(1,19) = 91.13$, $p < 0.001$, $\eta^2 = 0.83$]. A significant interaction of measurement time × training revealed fewer errors in trained than

untrained conditions after training [$F(1,19) = 6.19$, $p = 0.022$, $\eta^2 = 0.25$]. In order to explore training effects for simple and complex problems, two separate rmANOVAs were conducted for simple and complex multiplication. With respect to simple multiplication, no significant effect was found (cf. Fig. S3b). Regarding complex multiplication, a significant main effect of training [$F(1,19) = 17.09$, $p = 0.001$, $\eta^2 = 0.47$] was observed. A significant interaction effect of measurement time × training revealed that after training, children provided fewer errors in trained complex compared to untrained complex problems [$F(1,19) = 5.57$, $p = 0.029$, $\eta^2 = 0.23$]. Furthermore, a significant interaction of training × complexity was observed [$F(1,19) = 12.74$, $p = 0.002$, $\eta^2 = 0.40$].

FNIRS

Results from the whole measurement area for each measurement time

Furthermore, differences between trained and untrained conditions within each measurement time were investigated for fNIRS data. In the pre-training measurement, there was no significant difference in the contrast of trained simple versus untrained simple multiplication, or in the contrast of trained complex versus untrained complex (cf. Fig. S4a).

In the first post-training measurement, in the contrast of trained complex versus untrained complex multiplication, right SPL and IPS (channel 44) displayed significantly decreased activation [$t(19) = -2.52$, D/AP corrected $p < 0.05$, $d = 0.56$] (see Fig. S4b). Although reduced activation of the left AG (channel 14) and surrounding areas was observed, it did not survive correction for multiple statistical comparisons. No significant difference was found in the contrast of trained simple versus untrained simple multiplication.

In the two-week post-training measurement, in the contrast of trained complex versus untrained complex multiplication, the left MFG (channel 18) showed significantly decreased activation [$t(19) = -2.94$, D/AP corrected $p < 0.05$, $d = 0.66$] (cf. Fig. S4c). Although reduced activation of the left AG and STG (channel 5) was observed, this effect did not survive correction for multiple statistical comparisons. In the two-week post-training measurement, no significant difference between trained simple and untrained simple conditions was observed.

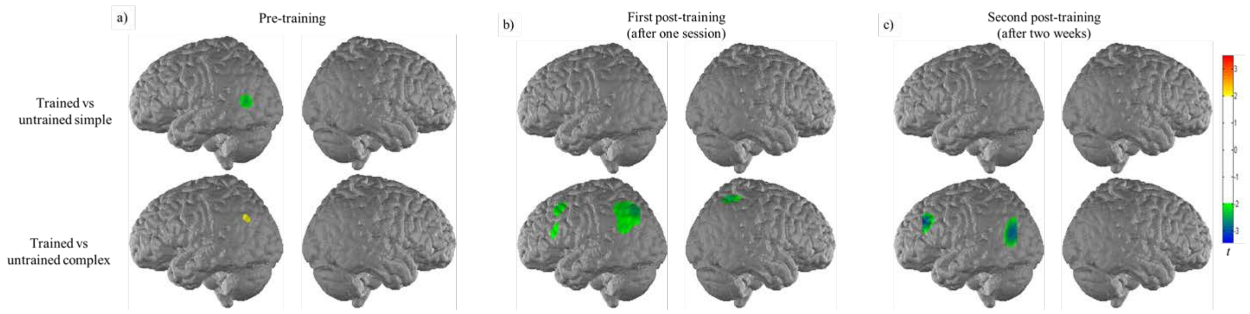

Fig. S4: a) FNIRS data showed no difference between trained and untrained conditions before the training. b) Although no effect of one-session training was observed in simple conditions, decreased activation of right SPL and IPS was found in trained complex compared to untrained complex multiplication. In the contrast of complex conditions, the huge deactivated area in the left parietal region did not survive correction for multiple comparisons. c) FNIRS data showed no two-week training effect in simple condition. The lower panel shows reduced activation of the left MFG for trained complex condition in the second post-training session after two weeks. In the contrast of complex conditions, the deactivated area in the left AG did not survive correction for multiple comparisons. The blue represents reduced activation, and the green represents non-significantly reduced activation.

EEG

Results from the whole measurement area for each measurement time

Regarding EEG, differences between trained and untrained conditions within each measurement time were investigated, the same as for fNIRS data. In the contrast of trained simple versus untrained simple multiplication in pre-training, greater theta ERS in the left temporal site (T7) [$t(19) = 2.29$, $p < 0.05$, $d = 0.51$], and lower theta ERS on the right frontal site (AFF4) [$t(19) = -2.30$, $p < 0.05$, $d = 0.51$], were observed (cf. Fig. S5a). No difference in alpha band in this contrast was demonstrated. In the contrast of trained complex versus untrained complex multiplication, no significant difference was found in the theta or alpha band (see Fig. S5a).

In the first post-training measurement, in the contrast of trained complex versus untrained complex multiplication, no significant difference was observed in the theta band, while in alpha band, greater alpha ERD on the occipito-parietal site (Pz, O2) was observed [$ts(19) < -2.10$, $ps < 0.05$, $ds > 0.47$] (cf. Fig. S5b). No significant difference was found in the contrast of trained simple versus untrained simple multiplication in the theta or alpha band (cf. Fig. S5b).

In the second post-training measurement, in the contrast of trained complex versus untrained complex multiplication, significantly decreased alpha ERD at the left occipital site (O1) was found [$t(19) = 2.85$, $p < 0.05$, $d = 0.64$]. In the contrast of trained simple versus untrained simple multiplication, an increased alpha ERD on the right temporal site (T8) [$t(19) = -2.17$, $p < 0.05$, $d = 0.49$], and a decreased alpha ERD on the right occipital site were observed (O2) [$t(19) = 2.20$, $p < 0.05$, $d = 0.49$]. No significant difference was found in the theta band in any of the contrasts (cf. Fig. S5c).

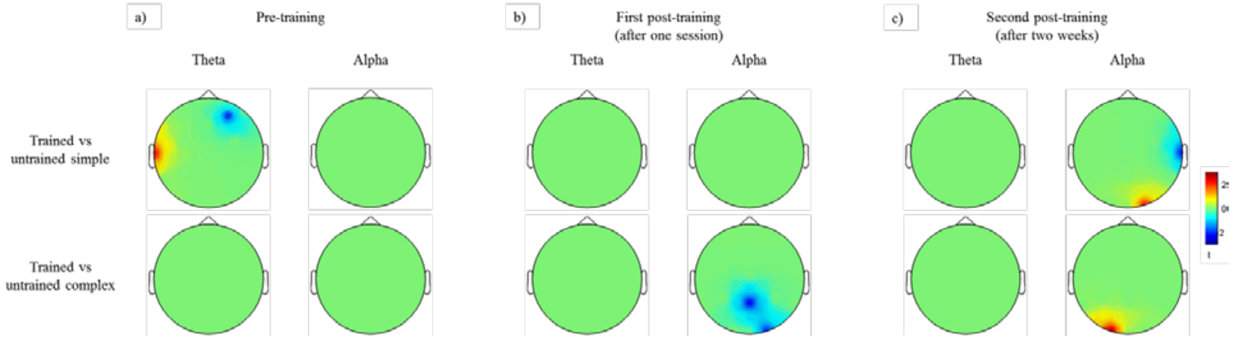

Fig. S5: a) Pre-training measurement showed no difference between trained and untrained conditions, except on theta band in the simple multiplication contrast. b) First post-training measurement shows no training effects in the simple condition, but increased alpha ERD in the trained complex compared to untrained complex multiplication. c) Alpha ERD changes were observed in both trained simple and complex conditions in the two-week post-training session. While no training change was observed in theta ERS, training led to changes in alpha ERD in both simple and complex multiplication. Red represents increased theta ERS/decreased alpha ERD, and blue represents decreased theta ERS/increased alpha ERD.

Correlation between behavioral performance and neuropsychological tests

In each measurement time, there were some significant correlations between performance factors including error rates, RTs, and inverse efficiency score with neuropsychological tests, especially verbal working memory in two-week measurement time (cf. Table S5).

Table S5: The correlation between error rates, RTs, and inverse efficiency scores at each measurement time with neuropsychological tests. The other performance measures were not correlated with any of neuropsychological tests.

Measurement time	Performance	Verbal IQ	Visuospatial IQ	Verbal STM	Verbal WM	Visuospatial STM	Visuospatial WM
Pre-training	US error rate	-.30	-.01	-.09	-.36	-.53*	-.64*
	US efficiency	-.28	-.07	.01	-.48*	-.36	-.44
First post-training	US error rate	-.34	-.21	-.21	-.45*	-.34	-.64*
Second post-training	TS RT	-.41	-.33	.19	-.53*	-.24	-.40
	TS efficiency	-.34	-.20	.15	-.47*	-.29	-.40
	TC RT	-.27	-.10	.13	-.51*	-.05	-.37
	TC efficiency	-.14	.01	.05	-.46*	.02	-.31
	US error rate	-.17	-.15	.17	-.54*	-.21	-.44
	US efficiency	-.17	-.08	.34	-.48*	-.26	-.40
	UC error rate	-.25	-.15	-.03	-.45*	-.01	-.30
	UC RT	-.48*	-.41	.20	-.34	-.36	-.65*
	UC efficiency	-.28	-.19	.16	-.49*	.03	-.25

Note: *$p < 0.05$, two-tailed; TS: trained simple; TC: trained complex; US: untrained simple; UC: untrained complex; STM: short-term memory; WM: working memory.

References cited in supplementary information

1 Huber, S., Fischer, U., Moeller, K. & Nuerk, H.-C. On the interrelation of multiplication and division in secondary school children. *Frontiers in psychology* **4** (2013).

2 Sankoh, A. J., Huque, M. F. & Dubey, S. D. Some comments on frequently used multiple endpoint adjustment methods in clinical trials. *Statistics in medicine* **16**, 2529-2542 (1997).

3 Bloechle, J. *et al.* Fact learning in complex arithmetic—the role of the angular gyrus revisited. *Human Brain Mapping* (2016).
